# Supplementary material for: Portuguese observational cross-sectional clinical imaging study protocol to investigate central dopaminergic mechanisms of successful weight loss through bariatric surgery
Source: BMJ Open. 2024 Apr 2;14(4):e080702. doi: 10.1136/bmjopen-2023-080702 (PMC10989096; doi:10.1136/bmjopen-2023-080702)
Supplement: Author's manuscript [file bmjopen-2023-080702.draft_revisions.pdf]

BMJ Open is committed to open peer review. As part of this commitment we make the peer review history of every article we publish publicly available.

When an article is published we post the peer reviewers' comments and the authors' responses online. We also post the versions of the paper that were used during peer review. These are the versions that the peer review comments apply to.

The versions of the paper that follow are the versions that were submitted during the peer review process. They are not the versions of record or the final published versions. They should not be cited or distributed as the published version of this manuscript.

BMJ Open is an open access journal and the full, final, typeset and author-corrected version of record of the manuscript is available on our site with no access controls, subscription charges or pay-per-view fees (http://bmjopen.bmj.com).

If you have any questions on BMJ Open's open peer review process please email info.bmjopen@bmj.com

BMJ Open

A clinical and molecular imaging study protocol to investigate central dopaminergic neurotransmission as a biosignature for obesity and treatment response in bariatric surgery

Journal:	*BMJ Open*
Manuscript ID	bmjopen-2023-080702
Article Type:	Protocol
Date Submitted by the Author:	08-Oct-2023
Complete List of Authors:	Pais, Marta Lapo; University of Coimbra<br>Crisóstomo, Joana; University of Coimbra<br>Abrunhosa, Antero; University of Coimbra<br>Castelo-Branco, Miguel; University of Coimbra
Keywords:	Bariatric Surgery, Obesity, Diagnostic Imaging, DIABETES & ENDOCRINOLOGY, RADIOLOGY & IMAGING

SCHOLARONE™
Manuscripts

Title

A clinical and molecular imaging study protocol to investigate central dopaminergic neurotransmission as a biosignature for obesity and treatment response in bariatric surgery

Authors

Marta **Lapo Pais**[a,b,d], Joana **Crisóstomo**[a,b], Antero **Abrunhosa**[a,b] and Miguel **Castelo-Branco**[a,b,c*]

[a] Coimbra Institute for Biomedical Imaging and Translational Research (CIBIT), University of Coimbra, Coimbra, Portugal

[b] Institute for Nuclear Sciences Applied to Health (ICNAS), University of Coimbra, Coimbra, Portugal

[c] Faculty of Medicine, University of Coimbra, Coimbra, Portugal

[d] Faculty of Science and Technology, University of Coimbra, Coimbra, Portugal

* Corresponding author (mcbranco@fmed.uc.pt)

For peer review only

ABSTRACT

Introduction

Bariatric surgery (BS) is the treatment of choice for refractory obesity. Although weight loss (WL) reduces the prevalence of obesity-related comorbidities and improves quality of life, not all patients achieve or maintain WL. Why some individuals are successful while others fail to respond remains a major research question. This protocol describes an observational study aiming to determine if central dopaminergic neurotransmission is unaltered in individuals who responded successfully to BS. As secondary goals, this protocol was also designed to investigate if dopaminergic status correlates with excess weight loss, total body weight loss and weight regain, and neurobehavioral and clinical parameters. Finally, we will also perform an exploratory analysis to investigate whether obesity and the type of BS (sleeve gastrectomy and Roux-en-Y gastric bypass) display distinct dopaminergic signatures and if they can be reversed after WL.

Methods and analysis

To address the main goal of this observational study, positron emission tomography (PET) with [^{11}C]Raclopride will be used to map brain dopamine type 2 and 3 receptors (D2/3R) of 21 individuals who have successfully responded to BS. Quantitative kinetic parameters will be extracted from these images and compared to 21 normal-weight healthy control individuals, with a focus on anatomical brain regions and fMRI identified regions of interest (ROIs) related to eating behaviour. The sample size of 42 was estimated assuming normally distributed groups for an effect size of d=0.8, $\alpha = 0.05$ and power of p=0.80. Exploratory analysis of PET and clinical data will use correlation /regression analysis. We expect to better understand the central mechanisms behind obesity and their impact on BS to identify the patients who will benefit from the procedure.

Ethics and dissemination

The project has received ethical approval from the ethics committee of the Faculty of Medicine of the University of Coimbra and *Centro Hospitalar e Universitário de Coimbra*.

Strengths and limitations of this study

→ Including an exploratory longitudinal analysis employing functional PET at two different time points, the study will be able to track BS-associated central dopaminergic neurotransmission changes;

→ Using functional brain regions related to eating behaviour (based on concomitant MRI data) will increase the statistical power of the study analysis by defining individualized regions of interest;

→ Investigating the central mechanisms of success in bariatric surgery will help identify the patients who will benefit from surgery;

→This study was designed to include only female participants;

→ Exploratory subgroup analysis is limited due to the dataset size, and in the longitudinal part of this analysis, some level of participant dropout is expected.

Abbreviations

For peer review only

BMI = Body Mass Index; BP_{ND} = nondisplaceable binding potential; BS = bariatric surgery; CHUC = *Centro Hospitalar e Universitário de Coimbra*; D2R = dopamine type 2 receptors; D2/3R = dopamine type 2 and 3 receptors; EWL% = excess weight loss; ICNAS = Institute for Nuclear Sciences Applied to Health; MRI = magnetic resonance imaging; PET = positron emission tomography; ROIs = regions of interest; WL% = total body weight loss; WL = weight loss.

INTRODUCTION

Background

Obesity is a multifactorial condition thought to result from gene-environment interactions and is mediated by complex neuronal and hormonal mechanisms.[1], [2] As a result, an imbalance of energy where energy intake exceeds energy expenditure leads to weight gain.[1], [2] This condition has become an epidemic of global proportions, being one of the leading causes of morbidity and mortality for the current and likely future generations.[3]

Bariatric surgery (BS) is the treatment of choice when other options have failed to treat obesity. Although weight loss (WL) reduces the prevalence of obesity-related comorbidities and improves the quality of life, not all patients manage to lose weight, and others regain it in the long term.[4]–[6] Why some individuals are successful while others have failed to respond to this therapy is an outstanding research question.

It has been suggested that WL after BS is likely due to changes in central nervous hunger and satiety control rather than purely restrictive gastric volume or gastrointestinal malabsorption.[7] This implies the existence of neural mechanisms underlying BS, and the role of central neurotransmission in hunger and satiety control.[8]–[11]Serotonin and dopamine have been considered the most important neurotransmitters in the homeostatic and reward systems, respectively.[8]–[10] Furthermore, two major "neurotransmitter hypotheses" of obesity postulate that increased food intake is partially driven by decreased dopamine-mediated reward or serotonin-mediated homeostatic feedback in response to food intake.[12]

Regarding overweight and obese phenotypes, the focus has been predominantly put on brain dopamine signaling.[13], [14] Concerning cerebral control of food intake, the

For peer review only

dopamine type 2 receptors (D2R) are particularly interesting because drugs that block D2R increase appetite and result in weight gain.[15] Furthermore, investigations involving human brain imaging and genetic analysis have revealed that individuals who are obese tend to exhibit modified D2R expression in some areas of the brain.[16] Yet, the role of brain dopamine receptors in overweight and obese phenotypes and success in BS and WL is neither consensual nor well addressed. For this reason, we propose to further investigate the role of these receptors in individuals with obesity and who have successfully responded to BS.

Study aims

This protocol describes an observational study exploring central dopaminergic neurotransmission as a biosignature of obese phenotypes and success in bariatric surgery. Namely, we will investigate central dopaminergic neurotransmission in individuals who have successfully responded to BS, i.e., who have at least 50% of excess weight loss (EWL%) or 20% of total body weight loss (WL%).

To achieve this main goal, positron emission tomography (PET) with [^{11}C]Raclopride will be used to map brain dopamine type 2 and 3 receptors (D2/3R) of 21 individuals who have successfully responded to BS. Quantitative kinetic parameters will be extracted from these images and compared to 21 normal-weight healthy control individuals, with a focus on anatomical brain regions and fMRI identified regions of interest (ROIs) related to eating behaviour.

Secondary questions will also be addressed using the same molecular imaging dopaminergic system, comparing groups of individuals (sleeve gastrectomy and Roux-en-Y gastric bypass, normal-weight and individuals with obesity and the same individuals before and after BS) and using correlation analysis. The correlation analysis will be used to determine if the dopaminergic system correlates with measures of BS treatment response, including EWL%, WL% and weight regain, and other key variables such as neurobehavioral, clinical parameters, and MRI measures (ROIs extracted in functional imaging tasks).

We expect that the proposed clinical and molecular imaging study will help to better understand brain central mechanisms of obesity and success in BS to ultimately identify

For peer review only

the patients who will benefit from surgery. Simultaneously a better understanding of the neural mechanisms of food motivation in overweight and obese phenotypes can contribute to the investigation and development of better non-invasive alternatives to treat obesity, which might have a wide impact in society and the quality of life of these individuals.

METHODS AND ANALYSIS

Participants

Two main groups of subjects will be included: individuals that have successfully responded to BS (21) recruited from *Centro Hospitalar e Universitário de Coimbra* (CHUC) or the Portuguese association of patients who underwent BS – *Associação Portuguesa dos Bariátricos* (APOBARI); and age- and gender-matched normal-weight healthy individuals (n= 21) recruited from our volunteer database and public advertisements. We will use the criteria that individuals submitted to BS with a successful treatment response present at least 50% of EWL% or 20% of WL% from 1 up to 5 years after BS.[17] For longitudinal exploratory analysis, we will include the same individuals in two different time points, before (individuals with obesity) and after BS. The study design and timeline are schematised in Figure 1.

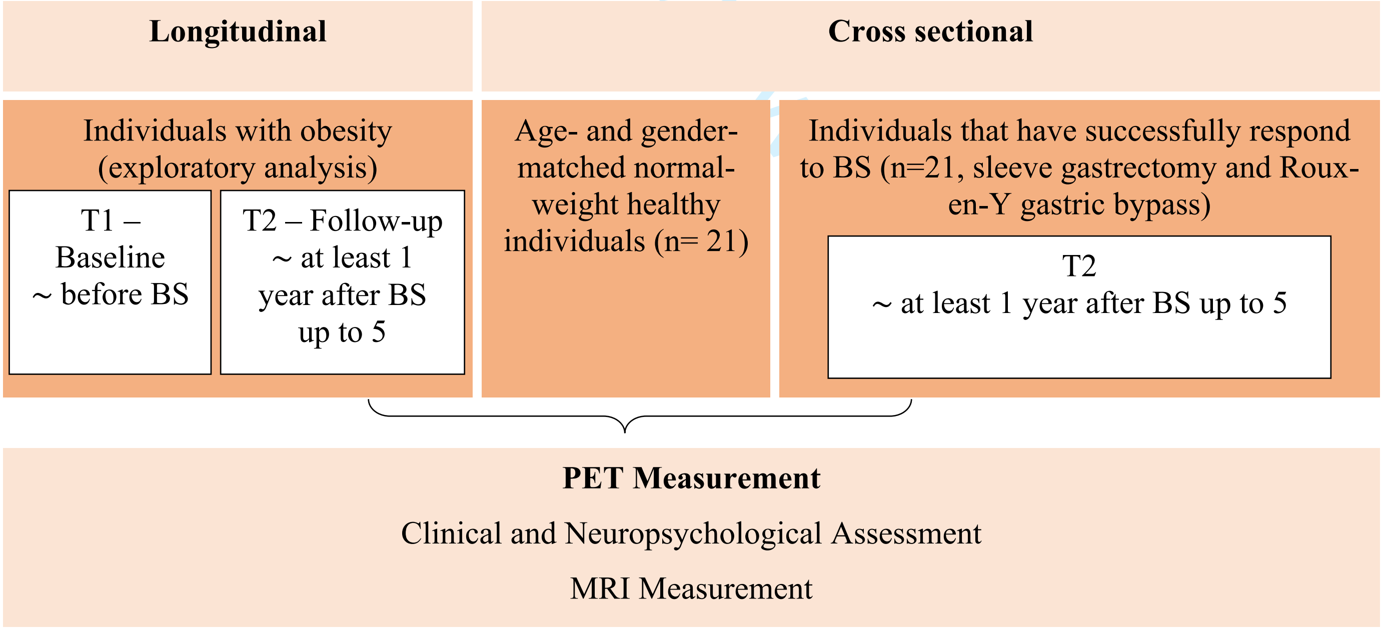

Figure 1 - Study design and timeline.

Study-specific 1) inclusion criteria: informed consent signed, women aged between 18 and 65 years, Body Mass Index (BMI) in the range of 35-50 kg/m2 for individuals with

For peer review only

obesity and 18.5-24.9 Kg/m2 for age- and gender-matched normal-weight healthy individuals; and 2) exclusion criteria: previous gastrointestinal surgery, current treatment with medication interacting with central dopamine, active neoplastic or inflammatory disease, history of neurological disease, traumatic brain injury or psychiatric disorder, previous/current alcohol or other substance abuse and conditions that preclude MRI or PET. All protocols were optimized by pilot acquisitions in healthy volunteers from our volunteer database.

Patient and public involvement

Patients were not involved in the development of the research question, outcome measures or study design, but were involved in the dissemination.

PET Measurement (acquisition and analysis)

Dynamic PET scan of the entire brain in 3D mode for 60 min (24 frames: 4x15sec, 4x30sec, 3x60sec, 2x120s, 5x240, 6x300s) together with computed tomography after intravenous bolus injection of a maximum of 15mCi of [^{11}C]Raclopride will be acquired using PET/CT scanner (Siemens Biograph Vision 600). Image acquisition and [^{11}C]Raclopride synthesis will be performed by a dedicated and experienced team in the facilities of the Institute for Nuclear Sciences Applied to Health (ICNAS) according to methods described in.[18]

After the acquisition, accurate co-registration (between PET images and their anatomical MRI images) will be performed and further confirmed by visual inspection across all planes. As binding of [^{11}C]Raclopride is negligible in the cerebellum and to avoid blood sampling, simplified reference tissue models will be used to analyse [^{11}C]Raclopride.[19] The outcome parameter of the cerebral [^{11}C]Raclopride binding will be the non-displaceable binding potential (BP_{ND}). BP_{ND} will be calculated using the cerebellum as a reference region. Two distinct reference tissue methods will be evaluated that are Logan Plot and MRTM2 (multilinear reference tissue model 2), as described and evaluated by the methods thoroughly reported in Logan (2000)[20] and Ichise et al. (2003)[21], respectively. All PET data analysis will be performed using the Pmod software (PMOD, version 4.105; PMOD Technologies; Zurich, Switzerland) and SPM12 (Version 12, Wellcome Trust Centre for Neuroimaging, London, UK).

For peer review only - http://bmjopen.bmj.com/site/about/guidelines.xhtml

Whole-brain and combined BP_{ND} values of several anatomical and functional brain ROIs will be carried out. ROIs will be defined in limbic, reward, and brain regions related to eating behaviour using masks of that regions or using the data from MRI that will be available in the same patients. Despite the lack of studies, we expect the same BP_{ND} values in individuals who successfully responded to BS and healthy controls. In exploratory analysis, it is expected that the obesity condition (BMI up to 50 kg/m^2) will be associated with unaltered dopamine D2/3R availability in the brain but increased for BMI greater than 50 kg/m^2. As described by previous studies using the same radiotracer.[22]–[24] Additionally, we expect that these altered BP_{ND} values can be reversed after WL.

Clinical and Neuropsychological Assessment

A brief neuropsychological evaluation will be performed together with the Eating Disorder Examination Questionnaire of 28 items[25], the Three Eating Factor Questionnaire of 21 items[26], the Self-Compassion Scale of 12 items[27] and the Depression Anxiety and Stress Scale of 21 items, all translated and validated for the Portuguese population. Studies propose that an emotional eating phenotype [28] or monetary reward discounting behaviour [29] might reflect altered central D2R function better than the commonly used BMI. [28], [29] As such we expect a negative correlation between measures of dysfunctional eating behaviour/concern and self-compassion, depression, anxiety, stress and WL after BS.

MRI Measurement (acquisition and analysis)

Structural and functional (visual task-based) MRI data (extracted in a concomitant tasks) will be acquired on a 3T imaging system (MAGNETOM Prisma, Siemens Medical Solutions) using a 64-channel head-coil. Briefly, stimuli will consist of the presentation of food images with high or low caloric categories, followed by a reported decision of whether eating or not such foods. The total time in the MRI scanner will not exceed 40 minutes. Image acquisition will be performed by dedicated and experienced technicians in the facilities of ICNAS.

As was mentioned before, fMRI data using visual stimuli will be used as localisers to map functional eating-related brain regions to use as ROIs in the quantitative analysis of PET brain images. Preprocessing such as head motion correction, mean intensity

For peer review only

adjustment, slice scan time correction, 3D motion correction and temporal filtering (high pass) will be performed using BrainVoyager 22.0.0 (Brain Innovation, Maastricht, The Netherlands). The same software can be used to perform statistical single-subject and group analyses.

Statistical analyses

Whole-brain and combined BP_{ND} values of several brain ROIs will be extracted from [^{11}C]Raclopride PET images of 21 individuals who have successfully responded to BS and compared to 21 normal-weight healthy control individuals, with a focus on anatomical brain regions and fMRI identified ROIs related to eating behaviour. Assuming normally distributed groups, statistically significant differences between these independent groups of individuals will be evaluated with the parametric two unpaired sample T-test (for equal variance) or Welch's t-test (for unequal variance). Non-parametric approaches will be taken if the data are not normally distributed according to the D'Agostino-Pearson's normality test.

On the other hand, the exploratory analysis will be qualitatively performed comparing groups of individuals to preliminary verify if obesity and the type of BS (sleeve gastrectomy and Roux-en-Y gastric bypass) display a distinct signature of brain dopamine and if altered dopaminergic neurotransmission can be reversed after WL. Finally, exploratory analysis of PET and clinical data will use correlation /regression analysis.

Sample size justification

The sample size was estimated in the G*Power 3.1.9.7 (Heinrich Heine University, Dusseldorf, Germany). Assuming normally distributed groups, we defined "t tests" as the test family, with "Means: difference between two independent means (two groups)" for between-group comparisons. For an allocation ratio of 1/1 (individuals submitted to BS Vs normal-weight), we obtain a total sample size of 42 (21 individuals submitted to BS and 21 normal-weight individuals) for an effect size of d=0.8, $\alpha = 0.05$ and power of p=0.80.

Final remarks

BS is the treatment of choice when other options have failed to treat obesity. However, the reason why some patients fail to respond to this therapy remains unclear. In this work

For peer review only

we will further investigate the role of dopaminergic neurotransmission in obesity and success in BS. Importantly, we propose to identify the brain maps of central dopamine receptors of success in BS and the neural basis of overweight, obese and WL.

To achieve the proposed goals we will map brain D2/3R of individuals who have successfully responded to BS, age and gender-matched normal-weight individuals and before and after BS using PET with [^{11}C]Raclopride. PET neuroimaging clinical studies which investigated the role of dopaminergic neurotransmission in obesity and BS treatment resistance and response are not consensual. For this reason, we propose to further investigate the role of these receptors in overweight and obese phenotypes, including individuals from normal-weight to obesity but also before and after bariatric surgery. Furthermore, to our knowledge, our study will be the first to characterise brain maps of central dopamine receptors of success in BS.

In summary, the findings of this work will hopefully clarify the role of dopaminergic D2/3R signalling in BS resistance, which would help identify the patients who will benefit from BS from those who may better be treated non-surgically. Simultaneously a better understanding of the neural basis of overweight and obese phenotypes and maintenance of WL can contribute to the investigation and development of better non-invasive alternatives to treat obesity, which would create a great societal impact and improvement in the quality of life of these individuals.

ETHICS AND DISSEMINATION

The project has received ethical approval from the local medical ethics committee of the Faculty of Medicine of the University of Coimbra (FMUC – approval ID, CE_Proc. CE-088/202) and *Centro Hospitalar e Universitário de Coimbra* (CHUC, approval ID OBS.SF.143/2021).

REFERENCES

[1] K. Pak, S. J. Kim, and I. J. Kim, "Obesity and Brain Positron Emission Tomography," *Nucl Med Mol Imaging*, vol. 52, no. 1, pp. 16–23, 2018, doi: 10.1007/s13139-017-0483-8.

[2] G. Ribeiro, M. Camacho, O. Santos, C. Pontes, S. Torres, and A. J. Oliveira-Maia, "Association between hedonic hunger and body-mass index versus obesity status," *Sci Rep*, vol. 8, no. 1, pp. 1–9, 2018, doi: 10.1038/s41598-018-23988-x.

For peer review only

[3] B. J. Sargent and A. J. Henderson, “Targeting 5-HT receptors for the treatment of obesity,” *Curr Opin Pharmacol*, vol. 11, no. 1, pp. 52–58, 2011, doi: 10.1016/j.coph.2011.01.005.
[4] M. C. Gulliford *et al.*, “Costs and outcomes of increasing access to bariatric surgery for obesity: cohort study and cost-effectiveness analysis using electronic health records,” *Health Services and Delivery Research*, vol. 4, no. 17, pp. 1–120, 2016, doi: 10.3310/hsdr04170.
[5] Z. Khorgami, S. Shoar, A. Andalib, A. Aminian, S. A. Brethauer, and P. R. Schauer, “Trends in utilization of bariatric surgery, 2010-2014: sleeve gastrectomy dominates,” *Surgery for Obesity and Related Diseases*, vol. 13, no. 5, pp. 774–778, 2017, doi: 10.1016/j.soard.2017.01.031.
[6] M. E. Haahr *et al.*, “Central 5-HT Neurotransmission Modulates Weight Loss following Gastric Bypass Surgery in Obese Individuals,” *Journal of Neuroscience*, vol. 35, no. 14, pp. 5884–5889, Apr. 2015, doi: 10.1523/JNEUROSCI.3348-14.2015.
[7] H. Schlögl, A. Horstmann, A. Villringer, and M. Stumvoll, “Functional neuroimaging in obesity and the potential for development of novel treatments,” *Lancet Diabetes Endocrinol*, vol. 4, no. 8, pp. 695–705, 2016, doi: 10.1016/S2213-8587(15)00475-1.
[8] M. K. Hankir, F. Seyfried, A. D. Miras, and M. A. Cowley, “Brain Feeding Circuits after Roux-en-Y Gastric Bypass,” *Trends in Endocrinology & Metabolism*, vol. 29, no. 4, pp. 218–237, Apr. 2018, doi: 10.1016/j.tem.2018.01.009.
[9] K. A. Galen, K. W. Horst, and M. J. Serlie, “Serotonin, food intake, and obesity,” *Obesity Reviews*, vol. 22, no. 7, pp. 1–13, Jul. 2021, doi: 10.1111/obr.13210.
[10] A. J. Oliveira-Maia, C. D. Roberts, S. A. Simon, and M. A. L. Nicolelis, “Gustatory and reward brain circuits in the control of food intake,” in *Psychiatry: Interpersonal and Biological Processes*, 2011, pp. 31–59. doi: 10.1007/978-3-7091-0179-7_3.
[11] M. A. Rossi and G. D. Stuber, “Overlapping Brain Circuits for Homeostatic and Hedonic Feeding,” *Cell Metab*, vol. 27, no. 1, pp. 42–56, Jan. 2018, doi: 10.1016/j.cmet.2017.09.021.
[12] K. A. van Galen, K. W. ter Horst, J. Booij, S. E. la Fleur, and M. J. Serlie, “The role of central dopamine and serotonin in human obesity: lessons learned from molecular neuroimaging studies,” *Metabolism*, vol. 85, pp. 325–339, 2018, doi: 10.1016/j.metabol.2017.09.007.
[13] S. Hesse *et al.*, “Central serotonin transporter availability in highly obese individuals compared with non-obese controls: A [11C] DASB positron emission tomography study,” *Eur J Nucl Med Mol Imaging*, vol. 43, no. 6, pp. 1096–1104, Jun. 2016, doi: 10.1007/s00259-015-3243-y.
[14] L. K. Janssen and A. Horstmann, “Molecular Imaging of Central Dopamine in Obesity: A Qualitative Review across Substrates and Radiotracers,” *Brain Sci*, vol. 12, no. 4, p. 486, Apr. 2022, doi: 10.3390/brainsci12040486.
[15] G.-J. Wang *et al.*, “Brain dopamine and obesity,” *The Lancet*, vol. 357, no. 9253, pp. 354–357, Feb. 2001, doi: 10.1016/S0140-6736(00)03643-6.
[16] J.-H. Baik, “Dopamine signaling in food addiction: role of dopamine D2 receptors,” *BMB Rep*, vol. 46, no. 11, pp. 519–526, Nov. 2013, doi: 10.5483/BMBRep.2013.46.11.207.
[17] S. Sanchez-Cordero *et al.*, “Analysis of the Variability in Different Criteria to Define the Success of Bariatric Surgery: Retrospective Study 5-Year Follow-Up

For peer review only

after Sleeve Gastrectomy and Roux-en-Y Gastric Bypass," *J Clin Med*, vol. 12, no. 1, p. 187, Dec. 2022, doi: 10.3390/jcm12010187.
[18] F. J. Alves, A. J. Abrunhosa, and I. B. Rodrigues, "Synthesis Optimisation of High Specific Activity Carbon-11 Radiopharmaceuticals for Brain PET Studies." Accessed: Feb. 20, 2023. [Online]. Available: https://eg.uc.pt/handle/10316/90183
[19] Turku PET entre, "Quantification of [carbonyl-11C]WAY-100635 PET." Accessed: Aug. 12, 2021. [Online]. Available: http://www.turkupetcentre.net/petanalysis/analysis_11c-way100635.html
[20] J. Logan, "Graphical analysis of PET data applied to reversible and irreversible tracers," *Nucl Med Biol*, vol. 27, no. 7, pp. 661–670, Oct. 2000, doi: 10.1016/S0969-8051(00)00137-2.
[21] M. Ichise *et al.*, "Linearized reference tissue parametric imaging methods: Application to [11C]DASB positron emission tomography studies of the serotonin transporter in human brain," *Journal of Cerebral Blood Flow and Metabolism*, vol. 23, no. 9, pp. 1096–1112, 2003, doi: 10.1097/01.WCB.0000085441.37552.CA.
[22] H. K. Karlsson *et al.*, "Obesity Is Associated with Decreased -Opioid But Unaltered Dopamine D2 Receptor Availability in the Brain," *Journal of Neuroscience*, vol. 35, no. 9, pp. 3959–3965, Mar. 2015, doi: 10.1523/JNEUROSCI.4744-14.2015.
[23] H. K. Karlsson *et al.*, "Weight loss after bariatric surgery normalizes brain opioid receptors in morbid obesity," *Mol Psychiatry*, vol. 21, no. 8, pp. 1057–1062, Aug. 2016, doi: 10.1038/mp.2015.153.
[24] L. Tuominen *et al.*, "Aberrant mesolimbic dopamine–opiate interaction in obesity," *Neuroimage*, vol. 122, pp. 80–86, Nov. 2015, doi: 10.1016/j.neuroimage.2015.08.001.
[25] P. P. P. Machado, C. Martins, A. R. Vaz, E. Conceição, A. P. Bastos, and S. Gonçalves, "Eating Disorder Examination Questionnaire: Psychometric Properties and Norms for the Portuguese Population," *European Eating Disorders Review*, vol. 22, no. 6, pp. 448–453, Nov. 2014, doi: 10.1002/erv.2318.
[26] P. A. S. Duarte, L. Palmeira, and J. Pinto-Gouveia, "The Three-Factor Eating Questionnaire-R21: a confirmatory factor analysis in a Portuguese sample," *Eating and Weight Disorders - Studies on Anorexia, Bulimia and Obesity*, vol. 25, no. 1, pp. 247–256, Feb. 2020, doi: 10.1007/s40519-018-0561-7.
[27] P. Castilho, J. Pinto-Gouveia, and J. Duarte, "Evaluating the Multifactor Structure of the Long and Short Versions of the Self-Compassion Scale in a Clinical Sample," *J Clin Psychol*, vol. 71, no. 9, pp. 856–870, Sep. 2015, doi: 10.1002/jclp.22187.
[28] S. A. Eisenstein *et al.*, "Emotional Eating Phenotype is Associated with Central Dopamine D2 Receptor Binding Independent of Body Mass Index," *Sci Rep*, vol. 5, no. 1, p. 11283, Sep. 2015, doi: 10.1038/srep11283.
[29] S. A. Eisenstein *et al.*, "Insulin, Central Dopamine D2 Receptors, and Monetary Reward Discounting in Obesity," *PLoS One*, vol. 10, no. 7, p. e0133621, Jul. 2015, doi: 10.1371/journal.pone.0133621.

AUTHORS' CONTRIBUTIONS

For peer review only

MLP, JC and MCB designed the protocol of the study. MLP and JC initiated the acquisition of data. MCB provided the funding for PET acquisition and AA provided the expertise for [^{11}C]Raclopride synthesis. MLP wrote the first draft of the manuscript. MLP, JC, AA and MCB critically revised the manuscript and approved the final version.

FUNDING

This Study Research Proposal was funded in 2020 by *Fundação para a Ciência e a Tecnologia* (FCT) for four years PhD Research Scholarship. Additional funding is secured by Coimbra Institute for Biomedical Imaging and Translational Research (CIBIT) Institutional Funding (FCT/UIDP&B/4950/2020), CENTRO-01-0145-FEDER-000016 and ICNAS/ICNAS Pharma.

COMPETING INTERESTS STATEMENT

The authors have no competing interests.

For peer review only

BMJ Open

An observational cross-sectional clinical imaging study protocol to investigate central dopaminergic mechanisms of successful weight loss through bariatric surgery

Journal:	*BMJ Open*
Manuscript ID	bmjopen-2023-080702.R1
Article Type:	Protocol
Date Submitted by the Author:	29-Dec-2023
Complete List of Authors:	Pais, Marta Lapo; University of Coimbra<br>Crisóstomo, Joana; University of Coimbra<br>Abrunhosa, Antero; University of Coimbra<br>Castelo-Branco, Miguel; University of Coimbra
**Primary Subject Heading**:	Diabetes and endocrinology
Secondary Subject Heading:	Radiology and imaging
Keywords:	Bariatric Surgery, Obesity, Diagnostic Imaging, DIABETES & ENDOCRINOLOGY, RADIOLOGY & IMAGING

SCHOLARONE™
Manuscripts

Title

An observational cross-sectional clinical imaging study protocol to investigate central dopaminergic mechanisms of successful weight loss through bariatric surgery

Authors

Marta Lapo Pais[a,b,d], Joana Crisóstomo[a,b], Antero Abrunhosa[a,b] and Miguel Castelo-Branco[a,b,c*]

[a] Coimbra Institute for Biomedical Imaging and Translational Research (CIBIT), University of Coimbra, Coimbra, Portugal

[b] Institute for Nuclear Sciences Applied to Health (ICNAS), University of Coimbra, Coimbra, Portugal

[c] Faculty of Medicine, University of Coimbra, Coimbra, Portugal

[d] Faculty of Science and Technology, University of Coimbra, Coimbra, Portugal

* Corresponding author (mcbranco@fmed.uc.pt)

ABSTRACT

Introduction

Bariatric surgery (BS) is the treatment of choice for refractory obesity. Weight loss (WL) reduces the prevalence of obesity-related comorbidities, but not all patients maintain it. It has been suggested that central mechanisms involving dopamine receptors may play a role in successful WL. This protocol describes an observational cross-sectional study to test if the binding of central dopamine receptors is similar in individuals who responded successfully to BS and age- and gender-matched normal-weight healthy individuals (controls). As secondary goals, the protocol will investigate if binding correlates with key parameters such as age, hormonal status, anthropometric metrics and neurobehavioral scores. As exploratory goals, we will include a cohort of individuals with obesity measured before and after BS. We will secondarily explore whether obesity and type of

For peer review only

BS (sleeve gastrectomy and Roux-en-Y gastric bypass) yield distinct binding values and track central dopaminergic changes resulting from BS.

Methods and analysis

To address the major research question of this observational study, positron emission tomography (PET) with [^{11}C]Raclopride will be used to map brain dopamine type 2 and 3 receptors (D2/3R) non-displaceable binding potential (BP_{ND}) of individuals who have successfully responded to BS. Mean regional D2/3R BP_{ND} values will be compared to 23 control individuals by two one-sided tests (TOST) approaches. This sample size (23 per group) was estimated to demonstrate the equivalence between two independent group means. Finally, BP_{ND} values will be correlated with key parameters to address secondary goals and compare within the same individuals (before and after BS) and between individuals with obesity and controls and types of BS for exploratory questions.

Ethics and dissemination

The project and informed consents received ethical approval from the ethics committees of the Faculty of Medicine (FMUC) and Coimbra University Hospital (CHUC). Results will be disseminated in international peer-reviewed journals and conferences.

Strengths and limitations of this study

→ Investigating brain dopamine receptors in successful WL through BS will ultimately help clarify how these receptors function in the context of maintenance of WL, decreased food intake and reduced motivational drive to eat after BS;

→ Including an exploratory longitudinal analysis employing functional PET at two different time points, the study will be able to track BS-associated central dopaminergic neurotransmission changes;

→ Using functionally defined brain regions related to eating behaviour will increase the statistical power of the study analysis by defining subject-specific activation clusters;

→This study was designed to include only female participants;

→ Exploratory subgroup analysis is limited due to the dataset size and, in the longitudinal part of this analysis, some level of participant dropout or exclusion is expected.

Abbreviations

For peer review only

BMI = Body Mass Index; BP_{ND} = nondisplaceable binding potential; BS = bariatric surgery; CHUC = Coimbra Hospital and University Centre; DASS= Depression Anxiety and Stress Scale; D2R = dopamine type 2 receptors; D2/3R = dopamine type 2 and 3 receptors; EDE-Q= Eating Disorder Examination Questionnaire; TE= echo time; EWL% = excess weight loss; FOV= field of view; FA= flip angle; fMRI = functional magnetic resonance imaging; ICNAS = Institute for Nuclear Sciences Applied to Health; MRI = magnetic resonance imaging; PET = positron emission tomography; TOST = two one-sided tests; TR= repetition time; ROIs = regions of interest; SCS= Self-Compassion Scale; TEFQ= Three Eating Factor Questionnaire; WL% = total body weight loss; WL = weight loss.

INTRODUCTION

Background

Obesity is a multifactorial condition thought to result from gene-environment interactions and is mediated by complex neuronal and hormonal mechanisms.[1], [2] As a result, an imbalance of energy where energy intake exceeds energy expenditure leads to weight gain.[1], [2] This condition has become an epidemic of global proportions, being one of the leading causes of morbidity and mortality for the current and likely future generations.[3]

Bariatric surgery (BS) is the treatment of choice when other options have failed to treat obesity. Although weight loss (WL) reduces the prevalence of obesity-related comorbidities and improves the quality of life, not all patients manage to lose weight, and others regain it in the long term.[4]–[6] It has been suggested that WL after BS is likely due to changes in central nervous hunger and satiety control rather than purely restrictive gastric volume or gastrointestinal malabsorption.[7] This implies the existence of neural mechanisms underlying BS, and the role of central neurotransmission in hunger and satiety control.[8]–[11] Dopamine is an important neurotransmitter with implications in addiction as well as WL, decreased food intake and a reduced motivational drive to eat.[12]–[15] Concerning cerebral control of food intake, the dopamine type 2 receptors (D2R) are particularly interesting because drugs that block D2R increase appetite and result in weight gain [16] and treatment with dopamine receptor agonists with a high affinity for D2R are reported to cause WL. [12] Moreover, a study found that dietary fat restriction significantly decreased D2R availability in striatal clusters of individuals with

For peer review only

obesity. [17] Yet, the role of brain dopamine receptors in eating behaviour that consequently impacts body weight, particularly their function in the context of successful WL through BS, is neither consensual nor well addressed. For this reason, we propose to further investigate these receptors in individuals who have successfully responded to BS.

Study aims

This protocol describes an observational cross-section study whose main goal is to investigate how brain dopamine type 2 and 3 receptors (D2/3R) function in the context of successful WL through BS. Namely, determine if the binding of these receptors is the same in individuals who responded successfully to BS and controls, age- and gender-matched normal-weight healthy individuals. To achieve that, positron emission tomography (PET) with [^{11}C]Raclopride will be used to map the binding of these receptors in 23 individuals of both groups. Several mean regional D2/3R non-displaceable binding potential (BP_{ND}) values will be extracted from these images, with a focus on three striatal brain regions (caudate, putamen and ventral striatum) and functional magnetic resonance imaging (fMRI) identified clusters related to eating behaviour.

As secondary goals, we aim to determine if these binding values correlate with anthropometric metrics, including waist/hip circumference, body mass index (BMI), excess weight loss (EWL%), total body weight loss (WL%) and weight regain, and other key variables such as neurobehavioral scores, age and hormonal status.

Finally, we will also perform an exploratory analysis using the same molecular imaging dopaminergic system. Comparing mean regional D2/3R BP_{ND} values of independent groups of individuals (sleeve gastrectomy and Roux-en-Y gastric bypass, controls and individuals with obesity) to investigate whether the type of BS and obesity display distinct dopamine receptors binding values, respectively; and comparing the same individuals before and after BS to track central dopaminergic changes resulting from BS.

We expect this work will help clarify how central mechanisms function in the context of maintenance of WL, decreased food intake and reduced motivational drive to eat after BS. Simultaneously exploring these central mechanisms in obese phenotypes and investigating the differences existing between types of BS can contribute to

For peer review only

understanding if the binding of these receptors depends on BMI or the type of surgery used.

METHODS AND ANALYSIS

Study design

This study can be split into two, main and secondary designs to answer the major and secondary and exploratory research questions, respectively. Both designs will be conducted at the Institute for Nuclear Sciences Applied to Health (ICNAS) University of Coimbra, Coimbra, Portugal. The expected overall study duration is approximately three years, from January 2022 (when we obtained both approvals from ethics committees) to January 2024. We anticipate a three-year timeframe, to disseminate the study in the community and social media, collect contacts of possible participants, optimise and test the study protocol by pilot acquisitions using healthy volunteers from our volunteer database, recruit participants, acquire, and analyse the data and submit the results for publication in peer-reviewed journals.

The main study design is an observational cross-sectional clinical imaging study to determine if the binding of D2/3R is the same in individuals who responded successfully to BS and controls. Two groups of female subjects will be included: individuals who have successfully responded to BS (n=23) recruited from Coimbra Hospital and University Centre (CHUC) or the Portuguese association of patients who underwent BS – *Associação Portuguesa dos Bariátricos* (APOBARI); and age- and gender-matched normal-weight (BMI: 18.5-24.9 Kg/m2) healthy individuals (n= 23) recruited from our volunteer database and public advertisements. To be considered successful, individuals should achieve at least 50% EWL% or 20% of WL% at 1 year post-op. [18] Since we will include participants from 1-5 years post-op, to verify this criterion we will use a retrospective approach and consider the weight participants had 1 year post-op. Additionally, we will also guarantee that at the time of data acquisition, participants still fulfil the criterion defined for 1 year after surgery (> 50% EWL% or 20% TWL%). Follow-up time post-op will be further used as a covariate in the statistical analysis.

The secondary study designs include a longitudinal and a cross-sectional part. The longitudinal part was designed to track central dopaminergic changes resulting from BS.

For peer review only

For that purpose, we will include a cohort of individuals with obesity at two different time points, before and after BS. On the other hand, in the cross-sectional part we will split the individuals who underwent BS according to the type of surgery to investigate whether the type of BS displays distinct dopamine receptor binding values. In addition, to investigate whether these binding values are altered in obesity we will also compare the individuals with obesity of the cohort with the controls. The study designs, research questions and samples are schematised in Figure 1.

Study-specific inclusion criteria are informed consent signed, women aged between 18 and 65 years, BMI >35 kg/m2 for individuals with obesity and 18.5-24.9 Kg/m2 for age- and gender-matched normal-weight healthy individuals. No restriction regarding BMI range will be imposed on the group who have successfully responded to BS. Exclusion criteria are previous gastrointestinal surgery, current treatment with medication interacting with central dopamine, active neoplastic or inflammatory disease, history of neurological disease, traumatic brain injury or psychiatric disorder, previous/current alcohol or other substance abuse and conditions that preclude MRI or PET.

Patient and public involvement

Patients were not involved in the development of the research question, outcome measures or study design, but were involved in the dissemination.

PET Measurement (acquisition and analysis)

Dynamic PET scan of the entire brain in 3D mode for 60 min (24 frames: 4x15sec, 4x30sec, 3x60sec, 2x120s, 5x240, 6x300s) together with computed tomography after intravenous bolus injection of a maximum of 15mCi of [^{11}C]Raclopride will be acquired using PET/CT scanner (Siemens Biograph Vision 600). [^{11}C]Raclopride synthesis will be performed by a dedicated and experienced team in the facilities of the ICNAS according to **the** methods described in**.[19]**

After acquisition, accurate co-registration (between PET images and their anatomical MRI images) will be performed and further confirmed by visual inspection across all planes. As binding of [^{11}C]Raclopride is negligible in the cerebellum and to avoid blood sampling, simplified reference tissue models will be used to analyse **PET images with**

For peer review only - http://bmjopen.bmj.com/site/about/guidelines.xhtml

[^{11}C]Raclopride.[20] As a result, we will obtain the parametric maps of D2/3R BP$_{ND}$. These maps will be generated using the cerebellum as a reference region and two distinct reference tissue methods will be evaluated that are Logan Plot and MRTM2 (multilinear reference tissue model 2), as described and evaluated by the methods thoroughly reported in Logan (2000)[21] and Ichise et al. (2003)[22], respectively. Mean regional D2/3R BP$_{ND}$ values of anatomical brain ROIs will be extracted from the parametric maps. Due to the low signal-to-noise ratio of [^{11}C]Raclopride in extrastriatal brain regions [23], anatomical ROIs will be defined in three subregions of the striatum, caudate, putamen and ventral striatum. Additionally, to increase the statistical power of the study analysis, we will also test subject-specific activation clusters that will result from fMRI analysis. All PET data analysis will be performed using the Pmod software (PMOD, version 4.105; PMOD Technologies; Zurich, Switzerland) and SPM12 (Version 12, Wellcome Trust Centre for Neuroimaging, London, UK).

Despite the lack of studies, we expect the same mean regional D2/3R BP$_{ND}$ values in individuals who have successfully responded to BS and healthy controls. We hypothesise that D2/3R BP$_{ND}$ depend on the motivation drive to eat, which we speculate to be the same in these two groups. Following the same line of thought, in exploratory analysis, we expect that obesity condition will be associated with altered mean regional D2/3R BP$_{ND}$ values in the brain but no differences between types of BS (sleeve gastrectomy and roux-en-Y gastric bypass). Moreover, we expect that these dysfunctional values can be reversed to control condition values after WL.

Clinical and Neuropsychological Assessment

The key variables of the clinical assessment include age, five anthropometric metrics and hormonal status. Anthropometric metrics are BMI, waist/hip circumference ratio for all groups and EWL%, WL% and weight regain for the group of individuals who underwent BS. As all our participants are female, and since dopaminergic neurotransmission fluctuates across age[24] and the menstrual cycle, this information will be further used as a covariate in the statistical analysis. A brief neuropsychological evaluation will also be performed together with the Eating Disorder Examination Questionnaire (EDE-Q) of 28 items[25], the Three Eating Factor Questionnaire (TEFQ) of 21 items[26], the Self-Compassion Scale (SCS) of 12 items[27] and the Depression Anxiety and Stress Scale (DASS) of 21 items, all translated and validated for the Portuguese population. EDE-Q

For peer review only

is categorised into 4 subscales (Restraint, Eating Concern, Shape Concern and Weight Concern) and an overall global score. TEFQ includes three factors (Cognitive Restraint, Uncontrolled eating and Emotional Eating). SCS is categorised into 6 subscales (self-kindness, self-judgement, common humanity, isolation, mindfulness and overidentification) and an overall global score. Finally, DASS is a set of three self-report scales designed to measure the emotional states of depression, anxiety and stress. To limit the number of parameters under investigation, for EDE-Q and SCS we will just include the overall global score.

Authors have suggested that emotional eating or behaviour of discounting monetary rewards might provide a more accurate reflection of changes in central D2R function compared to BMI [28], [29]. Following this line of thought and our hypothesis - D2/3R BP_{ND} depend on the motivation drive to eat - we expect that neuropsychological scores present a stronger correlation with central dopamine receptor function than anthropometric metrics. Namely, we expect to obtain a negative correlation between measures of dysfunctional eating behaviour/concern and self-compassion, depression, anxiety, stress and regional D2/3R BP_{ND} values and no correlation with anthropometric metrics.

MRI Measurement (acquisition and analysis)

Structural and functional MRI data will be acquired on a 3T imaging system (MAGNETOM Prisma, Siemens Medical Solutions) using a 64-channel head-coil. First, we will acquire the structural MRI data using 3D-T1 weighted sequence (192 slices; echo time (TE): 3.5 ms; repetition time (TR): 2530 ms; voxel size: 1×1×1 mm; flip angle (FA): 7°; field of view (FOV): 256×256 mm). After that, we will start the block-design fMRI paradigm (6 runs of ~2 minutes each) using 2D-T2* weighted sequences (72 interleaved slices; TE: 37ms; TR: 1000 ms; voxel size: 2×2×2 mm; FA: 68°; FOV: 200×200 mm). Each run comprised of three visual blocks and three question blocks begins with an instruction screen to test and set the directions (right and left) of the response buttons. The visual blocks have a duration of 20 seconds each and are composed of 5 pictures that change every 4 seconds. The first block is pictures of high or low caloric foods, the second is non-food objects and the third is the category of foods that did not appear in the first block. Before each visual block takes place a fixation cross (10 seconds) and after a question block (10 seconds), with a question about the visual block that took place before.

For peer review only - http://bmjopen.bmj.com/site/about/guidelines.xhtml

Food visual blocks will be followed by a decision-making question of whether the person would eat such foods, considering both the pleasure aspect and the health implications. To answer the question the participant will use a response button to choose one of four options: “1- I certainly wouldn't eat it”, “2- I probably wouldn't eat”, “3- I would probably eat” and “I would certainly eat it”. On the other hand, non-food visual blocks will be followed by an attention question of whether the person recognizes such objects. Here the options are the following: “1- I recognize all objects”, “2- I recognize almost all objects”; “3-I hardly recognize any object” and “4- I don't recognize any objects”. See Figure 2 for details.

As was mentioned before, fMRI data using visual stimuli will be used to obtain eating-related subject-specific activation clusters to use as masks in the quantitative analysis of PET brain images. Pre-processing of fMRI data includes head motion correction, mean intensity adjustment, cubic spine slice scan time correction, trilinear 3D motion correction and temporal filtering (high pass). Runs exceeding 3 mm of movement in any axis will be excluded from further analysis. After inhomogeneity correction of structural MRI data, functional and structural MRI data will be coregistered and transformed to the standard MNI space. Thereafter, a random effects General Linear Model analysis will be computed, for each subject, to assess the brain activity patterns. Finally, to obtain the eating-related subject-specific activation clusters we will use the conjunction contrast: [High & Low caloric foods > Objects] and Bonferroni correction to counteract the multiple comparisons with a fixed p-value of 0.05. All MRI data analysis will be performed using BrainVoyager 22.0.0 (Brain Innovation, Maastricht, The Netherlands).

Setting for imaging acquisition (PET and MRI)

PET and MRI acquisition will be performed in the same day by dedicated and experienced technicians in the facilities of ICNAS. Due to the well-documented impact of the fasted vs. fed state on responses to food cues, all subjects will perform MRI acquisition at the same time of the day, around 13:30 in a 6-hour (at least) fast state. To do so, participants will be instructed to have their last meal around 7:30. After MRI exam participants will consume the same standardized meal consisting of a vegetable soup, a sandwich, and fruit to ensure a similar recent dietary intake for all participants. For PET acquisition participants will be instructed to abstain from alcohol for 24 hours, as well as from nicotine and caffeine for 12 hours before this acquisition.

For peer review only

Statistical analyses

As already mentioned, mean regional BP_{ND} values will be extracted from the D2/3R BP_{ND} parametric maps of 23 individuals who have successfully responded to BS and 23 normal-weight healthy control individuals. To determine if the binding of these receptors is the same in these two groups, two one-sided tests (TOST) approaches will be performed. The TOST procedure entails performing two one-sided tests to examine whether the observed data is surprisingly larger than a lower equivalence boundary or surprisingly smaller than an upper equivalence boundary. To deal with multiple comparison issues, we will limit our hypotheses to three anatomical subregions of the striatum (caudate, putamen and ventral striatum) and one subject-specific activation cluster.

Since correlation is usually tested for two variables at a time, the association between the mean regional D2/3R BP_{ND} values with anthropometric, neurobehavioral and clinical parameters will lead us to the same multiple comparison issues. Since we aim to test several variables, we will perform a correlation matrix where the mean regional D2/3R BP_{ND} values of the three ROIs and one functional cluster will be correlated with the age, hormonal status, 5 anthropometric metrics, neuropsychological scores, and fMRI decision-making question responses. This will allow us to see which pairs have the highest correlation.

Finally, the exploratory analysis will be performed comparing independent groups of individuals to determine if obesity and the type of BS (sleeve gastrectomy and Roux-en-Y gastric bypass) display distinct mean regional D2/3R BP_{ND} values. Since we are comparing independent groups of individuals, we will use multiple unpaired t-test (for equal variance) or Welch's t-test (for unequal variance) followed by Holm-Sidak method (also used to deal with with multiple comparison issues). On the other hand, to track central dopaminergic changes resulting from BS, we will compare the same individuals before and after BS using ANOVA: Repeated measures, within-between interaction (for equal variance) or mixed-effects models (for unequal variance). In this case, to deal with multiple comparison issues we will perform Dunn's post hoc test.

Sample size justification

For peer review only - http://bmjopen.bmj.com/site/about/guidelines.xhtml

The sample size of 23 per group was estimated using the equation (5) published in Lakens (2017) as the required to demonstrate the equivalence between two independent group means for an allocation ratio of 1, equivalence bound of 0.8236, α level of 0.05 and power of 80%. [30] The equivalence bound of 0.8236 was calculated by multiplying 1.16 (Cohen's d) with 0.75 (pooled standard deviation). These values were set based on a meta-analysis performed to determine group differences in case–control studies comparing the same central dopamine receptors between individuals with obesity and non-obese controls. [31] In the case of significant differences, the overall Cohen's d was -1.16 $\pm$ 0.71.

ETHICS AND DISSEMINATION

The project has received ethical approval from the local medical ethics committee of the Faculty of Medicine of the University of Coimbra (FMUC – approval ID, CE_Proc. CE-088/202) and Coimbra Hospital and University Centre (CHUC, approval ID OBS.SF.143/2021). Participants will sign an informed consent approved by these ethics committees. Results will be submitted for publication in international peer-reviewed journals and presented at relevant conferences. Summaries of the results will be provided to the participants.

REFERENCES

[1] K. Pak, S. J. Kim, and I. J. Kim, "Obesity and Brain Positron Emission Tomography," *Nucl Med Mol Imaging*, vol. 52, no. 1, pp. 16–23, 2018, doi: 10.1007/s13139-017-0483-8.

[2] G. Ribeiro, M. Camacho, O. Santos, C. Pontes, S. Torres, and A. J. Oliveira-Maia, "Association between hedonic hunger and body-mass index versus obesity status," *Sci Rep*, vol. 8, no. 1, pp. 1–9, 2018, doi: 10.1038/s41598-018-23988-x.

[3] B. J. Sargent and A. J. Henderson, "Targeting 5-HT receptors for the treatment of obesity," *Curr Opin Pharmacol*, vol. 11, no. 1, pp. 52–58, 2011, doi: 10.1016/j.coph.2011.01.005.

[4] M. C. Gulliford *et al.*, "Costs and outcomes of increasing access to bariatric surgery for obesity: cohort study and cost-effectiveness analysis using electronic health records," *Health Services and Delivery Research*, vol. 4, no. 17, pp. 1–120, 2016, doi: 10.3310/hsdr04170.

[5] Z. Khorgami, S. Shoar, A. Andalib, A. Aminian, S. A. Brethauer, and P. R. Schauer, "Trends in utilization of bariatric surgery, 2010-2014: sleeve gastrectomy dominates," *Surgery for Obesity and Related Diseases*, vol. 13, no. 5, pp. 774–778, 2017, doi: 10.1016/j.soard.2017.01.031.

[6] M. E. Haahr *et al.*, "Central 5-HT Neurotransmission Modulates Weight Loss following Gastric Bypass Surgery in Obese Individuals," *Journal of*

For peer review only

Neuroscience, vol. 35, no. 14, pp. 5884–5889, Apr. 2015, doi: 10.1523/JNEUROSCI.3348-14.2015.

[7] H. Schlögl, A. Horstmann, A. Villringer, and M. Stumvoll, “Functional neuroimaging in obesity and the potential for development of novel treatments,” *Lancet Diabetes Endocrinol*, vol. 4, no. 8, pp. 695–705, 2016, doi: 10.1016/S2213-8587(15)00475-1.

[8] M. K. Hankir, F. Seyfried, A. D. Miras, and M. A. Cowley, “Brain Feeding Circuits after Roux-en-Y Gastric Bypass,” *Trends in Endocrinology & Metabolism*, vol. 29, no. 4, pp. 218–237, Apr. 2018, doi: 10.1016/j.tem.2018.01.009.

[9] K. A. Galen, K. W. Horst, and M. J. Serlie, “Serotonin, food intake, and obesity,” *Obesity Reviews*, vol. 22, no. 7, pp. 1–13, Jul. 2021, doi: 10.1111/obr.13210.

[10] A. J. Oliveira-Maia, C. D. Roberts, S. A. Simon, and M. A. L. Nicolelis, “Gustatory and reward brain circuits in the control of food intake,” in *Psychiatry: Interpersonal and Biological Processes*, 2011, pp. 31–59. doi: 10.1007/978-3-7091-0179-7_3.

[11] M. A. Rossi and G. D. Stuber, “Overlapping Brain Circuits for Homeostatic and Hedonic Feeding,” *Cell Metab*, vol. 27, no. 1, pp. 42–56, Jan. 2018, doi: 10.1016/j.cmet.2017.09.021.

[12] S. E. Simonds and M. A. Cowley, “Speed-dieting: dopamine agonists promote weight loss,” *Nat Metab*, vol. 1, no. 9, pp. 851–852, Aug. 2019, doi: 10.1038/s42255-019-0114-z.

[13] K. A. van Galen, K. W. ter Horst, J. Booij, S. E. la Fleur, and M. J. Serlie, “The role of central dopamine and serotonin in human obesity: lessons learned from molecular neuroimaging studies,” *Metabolism*, vol. 85, pp. 325–339, Aug. 2018, doi: 10.1016/j.metabol.2017.09.007.

[14] S. Hesse *et al.*, “Central serotonin transporter availability in highly obese individuals compared with non-obese controls: A [11C] DASB positron emission tomography study,” *Eur J Nucl Med Mol Imaging*, vol. 43, no. 6, pp. 1096–1104, Jun. 2016, doi: 10.1007/s00259-015-3243-y.

[15] L. K. Janssen and A. Horstmann, “Molecular Imaging of Central Dopamine in Obesity: A Qualitative Review across Substrates and Radiotracers,” *Brain Sci*, vol. 12, no. 4, p. 486, Apr. 2022, doi: 10.3390/brainsci12040486.

[16] G.-J. Wang *et al.*, “Brain dopamine and obesity,” *The Lancet*, vol. 357, no. 9253, pp. 354–357, Feb. 2001, doi: 10.1016/S0140-6736(00)03643-6.

[17] V. L. Darcey *et al.*, “Dietary fat restriction affects brain reward regions in a randomized crossover trial,” *JCI Insight*, vol. 8, no. 12, Jun. 2023, doi: 10.1172/jci.insight.169759.

[18] S. Sanchez-Cordero *et al.*, “Analysis of the Variability in Different Criteria to Define the Success of Bariatric Surgery: Retrospective Study 5-Year Follow-Up after Sleeve Gastrectomy and Roux-en-Y Gastric Bypass,” *J Clin Med*, vol. 12, no. 1, p. 187, Dec. 2022, doi: 10.3390/jcm12010187.

[19] F. J. Alves, A. J. Abrunhosa, and I. B. Rodrigues, “Synthesis Optimisation of High Specific Activity Carbon-11 Radiopharmaceuticals for Brain PET Studies.” Accessed: Feb. 20, 2023. [Online]. Available: https://eg.uc.pt/handle/10316/90183

[20] Turku PET entre, “Quantification of [carbonyl-11C]WAY-100635 PET.” Accessed: Aug. 12, 2021. [Online]. Available: http://www.turkupetcentre.net/petanalysis/analysis_11c-way100635.html

For peer review only

[21] J. Logan, "Graphical analysis of PET data applied to reversible and irreversible tracers," *Nucl Med Biol*, vol. 27, no. 7, pp. 661–670, Oct. 2000, doi: 10.1016/S0969-8051(00)00137-2.

[22] M. Ichise *et al.*, "Linearized reference tissue parametric imaging methods: Application to [11C]DASB positron emission tomography studies of the serotonin transporter in human brain," *Journal of Cerebral Blood Flow and Metabolism*, vol. 23, no. 9, pp. 1096–1112, 2003, doi: 10.1097/01.WCB.0000085441.37552.CA.

[23] T. Freiburghaus *et al.*, "Low convergent validity of [11C]raclopride binding in extrastriatal brain regions: A PET study of within-subject correlations with [11C]FLB 457," *Neuroimage*, vol. 226, p. 117523, Feb. 2021, doi: 10.1016/j.neuroimage.2020.117523.

[24] N. Karalija *et al.*, "Longitudinal Dopamine D2 Receptor Changes and Cerebrovascular Health in Aging," *Neurology*, vol. 99, no. 12, pp. e1278–e1289, Sep. 2022, doi: 10.1212/wnl.0000000000200891.

[25] P. P. P. Machado, C. Martins, A. R. Vaz, E. Conceição, A. P. Bastos, and S. Gonçalves, "Eating Disorder Examination Questionnaire: Psychometric Properties and Norms for the Portuguese Population," *European Eating Disorders Review*, vol. 22, no. 6, pp. 448–453, Nov. 2014, doi: 10.1002/erv.2318.

[26] P. A. S. Duarte, L. Palmeira, and J. Pinto-Gouveia, "The Three-Factor Eating Questionnaire-R21: a confirmatory factor analysis in a Portuguese sample," *Eating and Weight Disorders - Studies on Anorexia, Bulimia and Obesity*, vol. 25, no. 1, pp. 247–256, Feb. 2020, doi: 10.1007/s40519-018-0561-7.

[27] P. Castilho, J. Pinto-Gouveia, and J. Duarte, "Evaluating the Multifactor Structure of the Long and Short Versions of the Self-Compassion Scale in a Clinical Sample," *J Clin Psychol*, vol. 71, no. 9, pp. 856–870, Sep. 2015, doi: 10.1002/jclp.22187.

[28] S. A. Eisenstein *et al.*, "Insulin, Central Dopamine D2 Receptors, and Monetary Reward Discounting in Obesity," *PLoS One*, vol. 10, no. 7, p. e0133621, Jul. 2015, doi: 10.1371/journal.pone.0133621.

[29] S. A. Eisenstein *et al.*, "Emotional Eating Phenotype is Associated with Central Dopamine D2 Receptor Binding Independent of Body Mass Index," *Sci Rep*, vol. 5, no. 1, p. 11283, Sep. 2015, doi: 10.1038/srep11283.

[30] D. Lakens, "Equivalence Tests," *Soc Psychol Personal Sci*, vol. 8, no. 4, pp. 355–362, May 2017, doi: 10.1177/1948550617697177.

[31] G. Ribeiro, A. Maia, G. Cotovio, F. P. M. Oliveira, D. C. Costa, and A. J. Oliveira-Maia, "Striatal dopamine D2-like receptors availability in obesity and its modulation by bariatric surgery: a systematic review and meta-analysis," *Sci Rep*, vol. 13, no. 1, p. 4959, Mar. 2023, doi: 10.1038/s41598-023-31250-2.

AUTHORS' CONTRIBUTIONS

MLP, JC and MCB designed the protocol of the study. **JC and MCB designed the protocol of the MRI experiment.** MLP and JC initiated the acquisition of data. MCB supervised the project and provided the funding **for PET and MRI** acquisition and AA provided the expertise for [^{11}C]Raclopride synthesis. MLP wrote the first draft of the

For peer review only

manuscript. MLP, JC, AA and MCB critically revised the manuscript and approved the final version.

FUNDING

This Study Research Proposal was funded in 2020 by *Fundação para a Ciência e a Tecnologia* (FCT) for four years PhD Research Scholarship. Institutional funding is secured by Coimbra Institute for Biomedical Imaging and Translational Research (CIBIT) Funding (FCT/UIDP&B/4950/2020), CENTRO-01-0145-FEDER-000016 and ICNAS/ICNAS Pharma.

COMPETING INTERESTS STATEMENT

The authors have no competing interests.

Figure Legends

Figure 1 Main and secondary study designs, research questions and samples. Controls are the age- and gender-matched normal-weight healthy individuals.

Figure 2 Block-design representing one of the six runs of the fMRI paradigm. Each run begins with an instruction screen and is comprised of three visual blocks and three question blocks. The visual blocks have a duration of 20 seconds each and are composed by 5 pictures that change every 4 seconds. The first block is pictures of high or low caloric foods, the second is non-food objects and the third is the category of foods that did not appear in the first block. Before each visual block takes place a fixation cross (10 seconds) and after a question block (10 seconds), with a question about the visual block that took place before. Food visual blocks will be followed by a decision-making question of whether the person would eat such foods, considering both the pleasure aspect and the health implications. On the other hand, non-food visual blocks will be followed by an attention question of whether the person recognizes such objects.

For peer review only

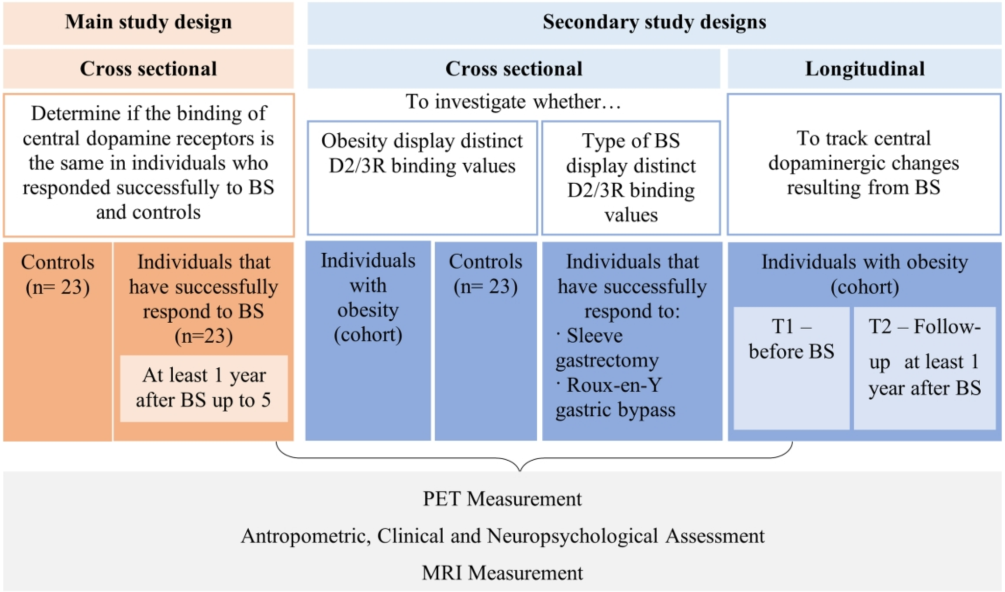

Figure 1 Main and secondary study designs, research questions and samples. Controls are the age- and gender-matched normal-weight healthy individuals.

197x119mm (300 x 300 DPI)

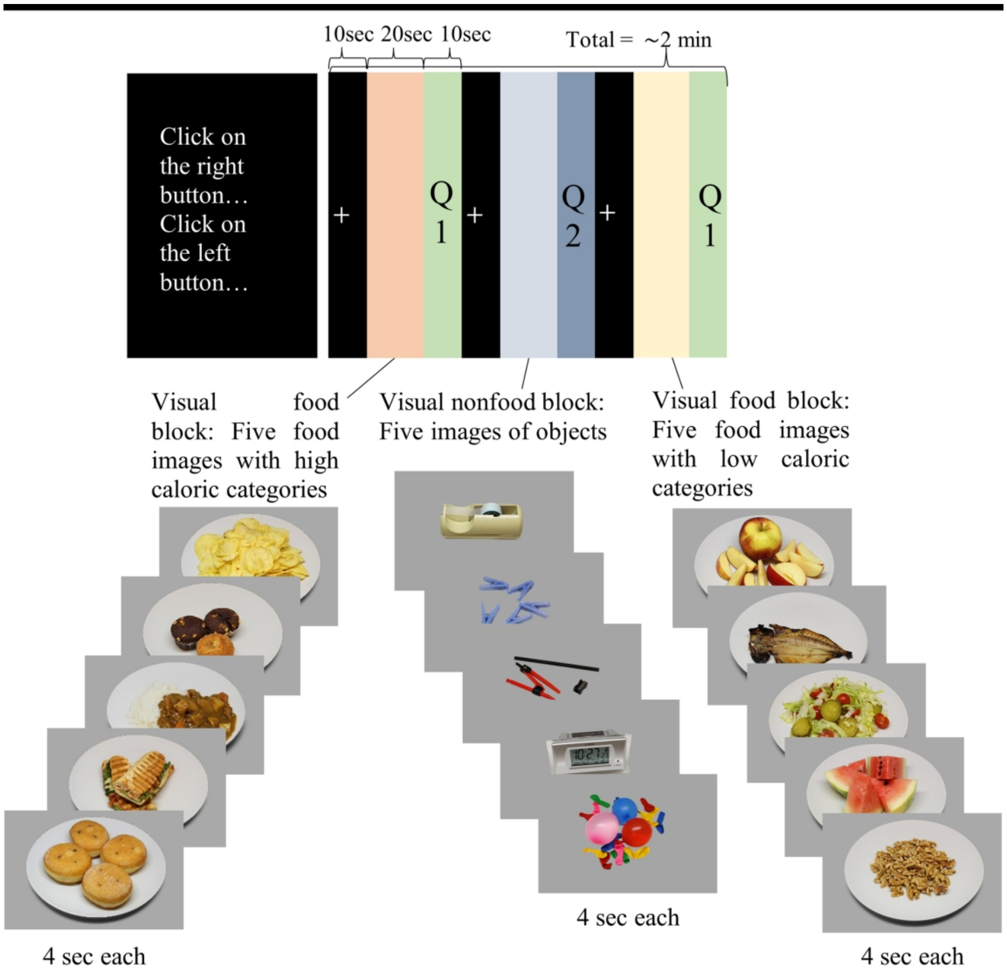

Figure 2 Block-design representing one of the six runs of the fMRI paradigm. Each run begins with an instruction screen and is comprised of three visual blocks and three question blocks. The visual blocks have a duration of 20 seconds each and are composed by 5 pictures that change every 4 seconds. The first block is pictures of high or low caloric foods, the second is non-food objects and the third is the category of foods that did not appear in the first block. Before each visual block takes place a fixation cross (10 seconds) and after a question block (10 seconds), with a question about the visual block that took place before. Food visual blocks will be followed by a decision-making question of whether the person would eat such foods, considering both the pleasure aspect and the health implications. On the other hand, non-food visual blocks will be followed by an attention question of whether the person recognizes such objects.

167x163mm (300 x 300 DPI)

BMJ Open

Portuguese observational cross-sectional clinical imaging study protocol to investigate central dopaminergic mechanisms of successful weight loss through bariatric surgery

Journal:	*BMJ Open*
Manuscript ID	bmjopen-2023-080702.R2
Article Type:	Protocol
Date Submitted by the Author:	12-Feb-2024
Complete List of Authors:	Pais, Marta Lapo; University of Coimbra<br>Crisóstomo, Joana; University of Coimbra<br>Abrunhosa, Antero; University of Coimbra<br>Castelo-Branco, Miguel; University of Coimbra
**Primary Subject Heading**:	Diabetes and endocrinology
Secondary Subject Heading:	Radiology and imaging
Keywords:	Bariatric Surgery, Obesity, Diagnostic Imaging, DIABETES & ENDOCRINOLOGY, RADIOLOGY & IMAGING

SCHOLARONE™
Manuscripts

Title

Portuguese observational cross-sectional clinical imaging study protocol to investigate central dopaminergic mechanisms of successful weight loss through bariatric surgery

Authors

Marta Lapo Pais[a,b,c], Joana Crisóstomo[a,d], Antero Abrunhosa[a,b] and Miguel Castelo-Branco[a,b,e*]

[a] Coimbra Institute for Biomedical Imaging and Translational Research (CIBIT), University of Coimbra, Coimbra, Portugal

[b] Institute for Nuclear Sciences Applied to Health (ICNAS), University of Coimbra, Coimbra, Portugal

[c] Faculty of Science and Technology, University of Coimbra, Coimbra, Portugal

[d] Champalimaud Research, Champalimaud Foundation, Lisbon, Portugal

[e] Faculty of Medicine, University of Coimbra, Coimbra, Portugal

* Corresponding author (mcbranco@fmed.uc.pt)

For peer review only

ABSTRACT

Introduction

Bariatric surgery (BS) is the treatment of choice for refractory obesity. Although weight loss (WL) reduces the prevalence of obesity-related comorbidities, not all patients maintain it. It has been suggested that central mechanisms involving dopamine receptors may play a role in successful WL. This protocol describes an observational cross-sectional study to test if the binding of central dopamine receptors is similar in individuals who responded successfully to BS and age- and gender-matched normal-weight healthy individuals (controls). As secondary goals, the protocol will investigate if this binding correlates with key parameters such as age, hormonal status, anthropometric metrics, and neurobehavioral scores. Finally, as exploratory goals, we will include a cohort of individuals with obesity before and after BS to explore whether obesity and type of BS

(sleeve gastrectomy and Roux-en-Y gastric bypass) yield distinct binding values and track central dopaminergic changes resulting from BS.

Methods and analysis

To address the major research question of this observational study, positron emission tomography (PET) with [^{11}C]raclopride will be used to map brain dopamine type 2 and 3 receptors (D2/3R) non-displaceable binding potential (BP_{ND}) of individuals who have successfully responded to BS. Mean regional D2/3R BP_{ND} values will be compared to control individuals by two one-sided tests (TOST) approaches. The sample size (23 per group) was estimated to demonstrate the equivalence between two independent group means. In addition, these binding values will be correlated with key parameters to address secondary goals. Finally, for exploratory analysis, these values will be compared within the same individuals (before and after BS) and between individuals with obesity and controls and types of BS.

Ethics and dissemination

The project and informed consent received ethical approval from the Faculty of Medicine (FMUC) and the Coimbra University Hospital (CHUC) ethics committees. Results will be disseminated in international peer-reviewed journals and conferences.

Strengths and limitations of this study

→ Investigating brain dopamine receptors in successful WL through BS will ultimately help clarify how these receptors function in the context of maintenance of WL, decreased food intake and reduced motivational drive to eat after BS;

→ Including an exploratory longitudinal analysis employing functional PET at two different time points, the study will be able to track BS-associated central dopaminergic neurotransmission changes;

→ Using functionally defined brain regions will increase the statistical power of the study analysis by defining a mean activation cluster related to visual food cue processing;

→This study was designed to include only female participants, which may limit the generalisability of our results.

→ Exploratory subgroup analysis is limited due to the dataset size, and some level of participant dropout or exclusion is expected in the longitudinal part of this analysis.

For peer review only

Abbreviations

BMI = Body Mass Index; BP_{ND} = nondisplaceable binding potential; BS = bariatric surgery; CHUC = Coimbra Hospital and University Centre; DASS= Depression Anxiety and Stress Scale; D2R = dopamine type 2 receptors; D2/3R = dopamine type 2 and 3 receptors; EDE-Q= Eating Disorder Examination Questionnaire; TE= echo time; EWL% = excess weight loss; FOV= field of view; FA= flip angle; fMRI = functional magnetic resonance imaging; GLM = General Linear Model; ICNAS = Institute for Nuclear Sciences Applied to Health; MRI = magnetic resonance imaging; PET = positron emission tomography; TOST = two one-sided tests; TR= repetition time; ROI = region of interest; SCS= Self-Compassion Scale; TEFQ= Three Eating Factor Questionnaire; WL% = total body weight loss; WL = weight loss.

INTRODUCTION

Background

Obesity is a multifactorial condition thought to result from gene-environment interactions and is mediated by complex neuronal and hormonal mechanisms.[1], [2] As a result, an imbalance of energy where energy intake exceeds energy expenditure leads to weight gain.[1], [2] This condition has become an epidemic of global proportions, being one of the leading causes of morbidity and mortality for the current and likely future generations.[3]

Bariatric surgery (BS) is the treatment of choice when other options have failed to treat obesity. Although weight loss (WL) reduces the prevalence of obesity-related comorbidities and improves the quality of life, not all patients manage to lose weight, and others regain it in the long term.[4], [5], [6] It has been suggested that WL after BS is likely due to changes in central nervous hunger and satiety control rather than purely restrictive gastric volume or gastrointestinal malabsorption.[7] This implies the existence of neural mechanisms underlying BS and the role of central neurotransmission in hunger and satiety control.[8], [9], [10], [11] Dopamine is an important neurotransmitter with implications in addiction as well as WL, decreased food intake and a reduced motivational drive to eat.[12], [13], [14], [15] Concerning cerebral control of food intake, the dopamine type 2 receptors (D2R) are particularly interesting. Original studies on this topic have been reporting that drugs that block D2R increase appetite and result in weight gain, such

For peer review only

as antipsychotic drugs[16] and treatment with dopamine receptor agonists with a high affinity for D2R causes WL.[17] Moreover, a study on diet manipulation found that dietary fat restriction significantly decreased D2R availability in striatal clusters of individuals with obesity.[18] Yet, the role of brain dopamine receptors in eating behaviour that consequently impacts body weight, particularly their function in the context of successful WL through BS, is neither consensual nor well addressed. For this reason, we propose to further investigate these receptors in individuals who have successfully responded to BS.

Study aims

This protocol describes an observational cross-section study whose main goal is to investigate how brain dopamine type 2 and 3 receptors (D2/3R) function in the context of successful WL through BS. Namely, we aim to understand if the binding of these receptors is the same in individuals who responded successfully to BS and controls, age- and gender-matched normal-weight healthy individuals. To achieve that, positron emission tomography (PET) with [^{11}C]raclopride will be used to map the binding of these receptors in 23 individuals of both groups. Mean regional D2/3R non-displaceable binding potential (BP_{ND}) values will be extracted from these images, with a focus on three striatal brain regions (caudate, putamen and ventral striatum) and a mean activation cluster across individuals related to visual food cue processing identified by functional magnetic resonance imaging (fMRI).

As secondary goals, we aim to evaluate if these binding values correlate with anthropometric metrics, including waist/hip circumference, body mass index (BMI), excess weight loss (EWL%), total body weight loss (WL%) and weight regain, and key variables such as neurobehavioral scores, age and hormonal status.

We will also perform an exploratory analysis using the same molecular imaging dopaminergic system. We will compare mean regional D2/3R BP_{ND} values of independent groups of individuals (sleeve gastrectomy and Roux-en-Y gastric bypass, controls and individuals with obesity) to investigate whether the type of BS and obesity display distinct dopamine receptors binding values, respectively. Finally, we will track central dopaminergic changes resulting from BS by comparing the same individuals before and after BS.

For peer review only

We expect this work will help clarify how central mechanisms function in the context of maintenance of WL, decreased food intake and reduced motivational drive to eat after BS. Simultaneously exploring these central mechanisms in obese phenotypes and investigating the differences between types of BS, we can contribute to understanding if the binding of these receptors depends on BMI or the type of surgery used.

METHODS AND ANALYSIS

Study design

This study can be split into main and secondary designs. The main design will be used to answer the major and secondary questions, whereas the secondary design will be used for exploratory research questions (see Figure 1). Both studies will be conducted at the Institute for Nuclear Sciences Applied to Health (ICNAS) University of Coimbra, Coimbra, Portugal. The expected overall study duration is approximately three years, from January 2022 (when we obtained both approvals from ethics committees) to January 2025. We anticipate a three-year timeframe to disseminate the study in the community and social media, collect contacts of possible participants, optimise and test the study protocol by pilot acquisitions using healthy volunteers from our volunteer database, recruit participants, acquire and analyse the data and submit the results for publication in peer-reviewed journals.

The main study design is an observational cross-sectional clinical imaging study to evaluate if the binding of D2/3R is the same in individuals who responded successfully to BS and controls. Two groups of female subjects will be included: women who have successfully responded to BS (n=23) recruited from Coimbra Hospital and University Centre (CHUC) or the Portuguese association of patients who underwent BS – *Associação Portuguesa dos Bariátricos* (APOBARI); and age- and gender-matched normal-weight (BMI: 18.5-24.9 Kg/m^2) healthy individuals (n= 23) recruited from our volunteer database and public advertisements. To be successful BS responders, individuals should achieve at least 50% EWL% or 20% of WL% at 1-year post-op.[19] Since we will include participants from 1-5 years post-op, we will use a retrospective approach to verify this criterion and consider the weight participants had 1-year post-op. We will also guarantee that at the time of data acquisition, participants still fulfil the

criterion defined for 1 year after surgery (> 50% EWL% or 20% TWL%). Follow-up time post-op will be further used as a covariate in the statistical analysis.

The secondary study design includes a longitudinal and a cross-sectional part. The longitudinal part was designed to track central dopaminergic changes resulting from BS. For that purpose, we will include a cohort of individuals with obesity at two different time points, before and after BS. On the other hand, in the cross-sectional part, we will split the individuals who underwent BS according to the type of surgery to investigate whether the type of BS displays distinct dopamine receptor binding values. Finally, to investigate whether these binding values are altered in obesity, we will compare the individuals with obesity of the cohort with the controls. The study designs, research questions, and samples are schematised in Figure 1.

Study-specific inclusion criteria are signed informed consent, women aged between 18 and 65 years, BMI >33 kg/m^2 for individuals with obesity and 18.5-24.9 Kg/m^2 for age- and gender-matched normal-weight healthy individuals. No restriction regarding BMI range will be imposed on the group who have successfully responded to BS. Exclusion criteria are previous gastrointestinal surgery, current treatment with medication interacting with central dopamine, active neoplastic or inflammatory disease, history of neurological disease, traumatic brain injury or psychiatric disorder, previous/current alcohol or other substance abuse and conditions that preclude magnetic resonance imaging (MRI) or PET.

Patient and public involvement

Patients were not involved in the development of the research question, outcome measures or study design but were involved in the dissemination.

PET Measurement (acquisition and analysis)

Dynamic PET scan of the entire brain in 3D mode for 60 min (24 frames: 4x15sec, 4x30sec, 3x60sec, 2x120s, 5x240, 6x300s) together with computed tomography after intravenous bolus injection of a maximum of 15mCi of [^{11}C]raclopride will be acquired using PET/CT scanner (Siemens Biograph Vision 600). A dedicated and experienced team will perform [^{11}C]raclopride synthesis in the facilities of the ICNAS according to the methods described in Rodrigues et al. (2020).[20]

For peer review only

After acquisition, accurate co-registration (between PET images and their anatomical MRI images) will be performed and further confirmed by visual inspection across all planes. As binding of [^{11}C]raclopride is negligible in the cerebellum, simplified reference tissue models will be used to analyse PET images with [^{11}C]raclopride.[21] As a result, we will obtain the parametric maps of D2/3R BP_{ND}. These maps will be generated using the cerebellum as a reference region, and two distinct reference tissue methods will be evaluated that are Logan Plot and MRTM2 (multilinear reference tissue model 2), as described and evaluated by the methods thoroughly reported in Logan (2000)[22] and Ichise et al. (2003)[23], respectively. Mean regional D2/3R BP_{ND} values of anatomical brain regions of interest (ROIs) will be extracted from the parametric maps. Due to the low signal-to-noise ratio of [^{11}C]raclopride in extrastriatal brain regions[24], anatomical ROIs will be defined in three subregions of the striatum, caudate, putamen and ventral striatum. Additionally, to increase the statistical power of the study analysis, we will test a mean activation cluster resulting from fMRI analysis, which will allow us to take into account other regions where dopaminergic receptors play an important biological role.[25] All PET data analysis will be performed using the Pmod software (PMOD, version 4.105; PMOD Technologies; Zurich, Switzerland) and SPM12 (Version 12, Wellcome Trust Centre for Neuroimaging, London, UK).

Despite the lack of studies, we expect the same mean regional D2/3R BP_{ND} values in individuals who have successfully responded to BS and healthy controls. We hypothesise that D2/3R BP_{ND} depend on the motivation drive to eat, which we speculate to be the same in these two groups. Following the same line of thought, in exploratory analysis, we expect that obesity will be associated with altered mean regional D2/3R BP_{ND} values in the brain but no differences between types of BS (sleeve gastrectomy and roux-en-Y gastric bypass). Moreover, we expect that these dysfunctional values can be reversed to control condition values after WL. It is important to note that because [^{11}C]raclopride is susceptible to endogenous dopamine, D2/3R BP_{ND} values do not reflect only receptor availability but also the levels of dopamine tone. A recent preprint study compared [^{11}C]raclopride to [^{18}F]fallypride, a radiotracer also susceptible to endogenous dopamine but with a higher affinity for D2/3R, and concluded that D2/3R BP_{ND} values measured by PET with [^{11}C]raclopride are more sensitive for revealing between-group differences resulting from dopamine tone.[26]

For peer review only - http://bmjopen.bmj.com/site/about/guidelines.xhtml

Clinical and Neuropsychological Assessment

The key variables of the clinical assessment include age, five anthropometric metrics and hormonal status. Anthropometric metrics are BMI, waist/hip circumference ratio for all groups and EWL%, WL% and weight regain for the group of individuals who underwent BS. As all our participants are female, and since dopaminergic neurotransmission might fluctuate across age[27] and the menstrual cycle[28], this information will be further used as a covariate in the statistical analysis. A brief neuropsychological evaluation will also be performed using the Eating Disorder Examination Questionnaire (EDE-Q) of 28 items[29], the Three Eating Factor Questionnaire (TEFQ) of 21 items[30], the Self-Compassion Scale (SCS) of 12 items[31] and the Depression Anxiety and Stress Scale (DASS) of 21 items, all translated and validated for the Portuguese population. EDE-Q has 4 subscales (Restraint, Eating Concern, Shape Concern and Weight Concern) and an overall global score. TEFQ includes three (Cognitive Restraint, Uncontrolled Eating and Emotional Eating). SCS is split into 6 subscales (Self-kindness, Self-judgement, Common Humanity, Isolation, Mindfulness and Overidentification) and an overall global score. Finally, DASS measure the emotional states of depression, anxiety and stress. To limit the number of parameters under investigation, for EDE-Q and SCS, we will only include the overall global score.

Authors have suggested that emotional eating or behaviour of discounting monetary rewards might provide a more accurate reflection of changes in central D2R function compared to BMI[32], [33]. Following this line of thought and our hypothesis - D2/3R BP_{ND} depend on the motivation drive to eat - we expect that neuropsychological scores correlate more strongly with central dopamine receptor function than anthropometric metrics. Namely, we expect to obtain a negative correlation between measures of dysfunctional eating behaviour/concern and self-compassion, depression, anxiety, stress and regional D2/3R BP_{ND} values and no correlation with anthropometric metrics.

MRI Measurement (acquisition and analysis)

Structural and fMRI data will be acquired on a 3T imaging system (MAGNETOM Prisma, Siemens Medical Solutions) using a 64-channel head-coil. First, we will acquire the structural MRI data using 3D-T1 weighted sequence (192 slices; echo time (TE): 3.5 ms; repetition time (TR): 2530 ms; voxel size: 1×1×1 mm; flip angle (FA): 7°; field

For peer review only

of view (FOV): 256×256 mm). After that, we will start the block-design fMRI paradigm (6 runs of ~2 minutes each) using 2D-T2* weighted sequences (72 interleaved slices; TE: 37ms; TR: 1000 ms; voxel size: 2×2×2 mm; FA: 68°; FOV: 200×200 mm). Each run comprises three visual blocks and three question blocks. In each visual block (20 seconds), participants will be presented with 5 images of low or high-calorie foods or non-food objects from the Cross Cultural Food Images Database (CROCUFID).[34] For all participants, the sequence of the fMRI task is the same, where the first block switches between high- and low-calorie foods, while the block of non-food objects is interleaved in the middle. Before each visual block takes place a fixation cross (10 seconds) and after a question block (10 seconds), with a question about the visual block that took place before. Food visual blocks will be followed by a decision-making question of whether the person would eat such foods, considering both the pleasure and health implications. To answer the question, the participant will use a response button to choose one of four options: "1- I certainly wouldn't eat it", "2- I probably wouldn't eat", "3- I would probably eat" and "I would certainly eat it". On the other hand, non-food visual blocks will be followed by an attention question of whether the person recognises such objects. Here, the options are the following: "1- I recognise all objects", "2- I recognise almost all objects"; "3-I hardly recognise any object" and "4- I don't recognise any objects". See Figure 2 for details. We plan to use the decision-making questions to evaluate how people rate rewards in terms of pleasure and health concerns. Namely, achieve information on dissociable psychological components of reward, particularly liking (hedonic impact) and wanting (incentive salience). This information will be combined with the neuropsychological scores to conduct a correlation analysis with central dopamine receptor function. Finally, after the fMRI acquisition, the participants will also perform rating tasks using a horizontal, 100-mm, bidirectional scale between the extremes "I don't like it at all" and "I like it a lot" to rate all the food images from fMRI visual task in terms of preferences regardless of health concerns. This information will also be used as a variable in the correlation analysis with central dopamine receptor function.

As was mentioned before, fMRI data using visual stimuli will be used to obtain a mean activation cluster related to visual food cue processing to use as mask in the quantitative analysis of PET brain images. Pre-processing of fMRI data includes head motion correction, mean intensity adjustment, cubic spine slice scan time correction, trilinear 3D motion correction and temporal filtering (high pass). Runs exceeding 3 mm of movement

For peer review only

in any axis will be excluded from further analysis. After inhomogeneity correction of structural MRI data, functional and structural MRI data will be coregistered and transformed to the standard Montreal Neurological Institute (MNI) space. A random effects General Linear Model (GLM) analysis will be computed for each subject to assess individual brain activity patterns. After that, to obtain activation clusters related to visual food cue processing, we will use contrasts between [High-calorie], [Low-calorie] and [Non-food objects]. Finally, we will aggregate the individual activation clusters in a mean activation cluster across groups to use as a mask in the PET analysis. Due to the low signal-to-noise ratio of [^{11}C]raclopride in extrastriatal brain regions[24], we plan to be cautious while not ignoring that D2R are present with meaningful biological function outside the striatum.[25] All MRI data analysis will be performed using BrainVoyager 22.0.0 (Brain Innovation, Maastricht, The Netherlands).

Setting for imaging acquisition (PET and MRI)

PET and MRI acquisition will be performed on the same day by dedicated and experienced technicians in the facilities of ICNAS. Due to the well-documented impact of the fasted vs. fed state on responses to food cues, all subjects will perform MRI acquisition at the same time of the day, around 13:30 in a 6-hour (at least) fast state. To do so, participants will be instructed to have their last meal around 7:30. After the MRI scan, participants will consume the same standardised meal consisting of a vegetable soup, a sandwich, and fruit to ensure a similar recent dietary intake for all participants. For PET acquisition, participants will be instructed to abstain from alcohol for 24 hours, as well as from nicotine and caffeine for 12 hours before this acquisition.

Statistical analyses

As already mentioned, mean regional binding values will be extracted from the D2/3R BP_{ND} parametric maps of 23 individuals who have successfully responded to BS and 23 normal-weight healthy control individuals. Two one-sided tests (TOST) approaches will be performed to investigate if the binding of these receptors is the same in these two groups. To deal with multiple comparison issues, we will limit our hypotheses to three anatomical subregions of the striatum (caudate, putamen and ventral striatum) and a mean activation cluster across groups.

For peer review only

Since correlation is usually tested for two variables at a time, the association between the mean regional D2/3R BP_{ND} values with anthropometric, neurobehavioral and clinical parameters will lead us to the same multiple comparison issues. Since we aim to test several variables, we will perform a correlation matrix where the mean regional D2/3R BP_{ND} values of the three ROIs and the mean activation cluster across groups will be correlated with the age, hormonal status, 5 anthropometric metrics, neuropsychological scores, and fMRI decision-making question responses. The generated matrix will allow us to see which pairs correlate most.

Finally, the exploratory analysis will compare independent groups of individuals to verify if obesity and the type of BS (sleeve gastrectomy and Roux-en-Y gastric bypass) display distinct mean regional D2/3R BP_{ND} values. Since we are comparing independent groups of individuals, we will use multiple unpaired t-tests (for equal variance) or Welch's t-test (for unequal variance) followed by the Holm-Sidak method (to deal with multiple comparison issues). On the other hand, to track central dopaminergic changes resulting from BS, we will compare the same individuals before and after BS using ANOVA: Repeated measures, within-between interaction (for equal variance) or mixed-effects models (for unequal variance). We will perform Dunn's post hoc test to deal with multiple comparison issues in this case.

Sample size justification

The sample size of 23 per group was estimated using the equation (5) published in Lakens (2017) as the required to demonstrate the equivalence between two independent group means for an allocation ratio of 1, equivalence bound of 0.8236, α level of 0.05 and power of 80%.[35] The equivalence bound of 0.8236 was calculated by multiplying 1.16 (Cohen's d) with 0.71 (pooled standard deviation). These values were set based on a meta-analysis performed to investigate group differences in case–control studies comparing the same central dopamine receptors between individuals with obesity and non-obese controls.[36] In the case of significant differences, the overall Cohen's d was -1.16 $\pm$ 0.71.

ETHICS AND DISSEMINATION

The project has received ethical approval from the local medical ethics committee of the Faculty of Medicine of the University of Coimbra (FMUC – approval ID, CE_Proc. CE-

For peer review only

088/202) and Coimbra Hospital and University Centre (CHUC, approval ID OBS.SF.143/2021). Participants will sign an informed consent approved by these ethics committees. Results will be submitted for publication in international peer-reviewed journals and presented at relevant conferences. Summaries of the results will be provided to the participants.

REFERENCES

[1] K. Pak, S. J. Kim, and I. J. Kim, "Obesity and Brain Positron Emission Tomography," *Nucl Med Mol Imaging*, vol. 52, no. 1, pp. 16–23, 2018, doi: 10.1007/s13139-017-0483-8.

[2] G. Ribeiro, M. Camacho, O. Santos, C. Pontes, S. Torres, and A. J. Oliveira-Maia, "Association between hedonic hunger and body-mass index versus obesity status," *Sci Rep*, vol. 8, no. 1, pp. 1–9, 2018, doi: 10.1038/s41598-018-23988-x.

[3] B. J. Sargent and A. J. Henderson, "Targeting 5-HT receptors for the treatment of obesity," *Curr Opin Pharmacol*, vol. 11, no. 1, pp. 52–58, 2011, doi: 10.1016/j.coph.2011.01.005.

[4] M. C. Gulliford *et al.*, "Costs and outcomes of increasing access to bariatric surgery for obesity: cohort study and cost-effectiveness analysis using electronic health records," *Health Services and Delivery Research*, vol. 4, no. 17, pp. 1–120, 2016, doi: 10.3310/hsdr04170.

[5] Z. Khorgami, S. Shoar, A. Andalib, A. Aminian, S. A. Brethauer, and P. R. Schauer, "Trends in utilization of bariatric surgery, 2010-2014: sleeve gastrectomy dominates," *Surgery for Obesity and Related Diseases*, vol. 13, no. 5, pp. 774–778, 2017, doi: 10.1016/j.soard.2017.01.031.

[6] M. E. Haahr *et al.*, "Central 5-HT Neurotransmission Modulates Weight Loss following Gastric Bypass Surgery in Obese Individuals," *Journal of Neuroscience*, vol. 35, no. 14, pp. 5884–5889, Apr. 2015, doi: 10.1523/JNEUROSCI.3348-14.2015.

[7] H. Schlögl, A. Horstmann, A. Villringer, and M. Stumvoll, "Functional neuroimaging in obesity and the potential for development of novel treatments," *Lancet Diabetes Endocrinol*, vol. 4, no. 8, pp. 695–705, 2016, doi: 10.1016/S2213-8587(15)00475-1.

[8] M. K. Hankir, F. Seyfried, A. D. Miras, and M. A. Cowley, "Brain Feeding Circuits after Roux-en-Y Gastric Bypass," *Trends in Endocrinology & Metabolism*, vol. 29, no. 4, pp. 218–237, Apr. 2018, doi: 10.1016/j.tem.2018.01.009.

[9] K. A. Galen, K. W. Horst, and M. J. Serlie, "Serotonin, food intake, and obesity," *Obesity Reviews*, vol. 22, no. 7, pp. 1–13, Jul. 2021, doi: 10.1111/obr.13210.

[10] A. J. Oliveira-Maia, C. D. Roberts, S. A. Simon, and M. A. L. Nicolelis, "Gustatory and reward brain circuits in the control of food intake," in *Psychiatry: Interpersonal and Biological Processes*, 2011, pp. 31–59. doi: 10.1007/978-3-7091-0179-7_3.

[11] M. A. Rossi and G. D. Stuber, "Overlapping Brain Circuits for Homeostatic and Hedonic Feeding," *Cell Metab*, vol. 27, no. 1, pp. 42–56, Jan. 2018, doi: 10.1016/j.cmet.2017.09.021.

For peer review only

[12] S. E. Simonds and M. A. Cowley, "Speed-dieting: dopamine agonists promote weight loss," *Nat Metab*, vol. 1, no. 9, pp. 851–852, Aug. 2019, doi: 10.1038/s42255-019-0114-z.
[13] K. A. van Galen, K. W. ter Horst, J. Booij, S. E. la Fleur, and M. J. Serlie, "The role of central dopamine and serotonin in human obesity: lessons learned from molecular neuroimaging studies," *Metabolism*, vol. 85, pp. 325–339, Aug. 2018, doi: 10.1016/j.metabol.2017.09.007.
[14] S. Hesse *et al.*, "Central serotonin transporter availability in highly obese individuals compared with non-obese controls: A [11C] DASB positron emission tomography study," *Eur J Nucl Med Mol Imaging*, vol. 43, no. 6, pp. 1096–1104, Jun. 2016, doi: 10.1007/s00259-015-3243-y.
[15] L. K. Janssen and A. Horstmann, "Molecular Imaging of Central Dopamine in Obesity: A Qualitative Review across Substrates and Radiotracers," *Brain Sci*, vol. 12, no. 4, p. 486, Apr. 2022, doi: 10.3390/brainsci12040486.
[16] M.-J. Poulin et al., "Management of Antipsychotic-Induced Weight Gain: Prospective Naturalistic Study of the Effectiveness of a Supervised Exercise Programme," Australian & New Zealand Journal of Psychiatry, vol. 41, no. 12, pp. 980–989, Dec. 2007, doi: 10.1080/00048670701689428.
[17] C. Folgueira *et al.*, "Hypothalamic dopamine signalling regulates brown fat thermogenesis," *Nat Metab*, vol. 1, no. 8, pp. 811–829, Aug. 2019, doi: 10.1038/s42255-019-0099-7.
[18] V. L. Darcey *et al.*, "Dietary fat restriction affects brain reward regions in a randomized crossover trial," *JCI Insight*, vol. 8, no. 12, Jun. 2023, doi: 10.1172/jci.insight.169759.
[19] S. Sanchez-Cordero *et al.*, "Analysis of the Variability in Different Criteria to Define the Success of Bariatric Surgery: Retrospective Study 5-Year Follow-Up after Sleeve Gastrectomy and Roux-en-Y Gastric Bypass," *J Clin Med*, vol. 12, no. 1, p. 187, Dec. 2022, doi: 10.3390/jcm12010187.
[20] I. Beatriz, A. Rodrigues, F. José, and C. Alves, "Synthesis Optimisation of High Specific Activity Carbon-11 Radiopharmaceuticals for Brain PET Studies," 2020.
[21] Turku PET entre, "Quantification of [carbonyl-11C]WAY-100635 PET." Accessed: Aug. 12, 2021. [Online]. Available: http://www.turkupetcentre.net/petanalysis/analysis_11c-way100635.html
[22] J. Logan, "Graphical analysis of PET data applied to reversible and irreversible tracers," *Nucl Med Biol*, vol. 27, no. 7, pp. 661–670, Oct. 2000, doi: 10.1016/S0969-8051(00)00137-2.
[23] M. Ichise *et al.*, "Linearized reference tissue parametric imaging methods: Application to [11C]DASB positron emission tomography studies of the serotonin transporter in human brain," *Journal of Cerebral Blood Flow and Metabolism*, vol. 23, no. 9, pp. 1096–1112, 2003, doi: 10.1097/01.WCB.0000085441.37552.CA.
[24] T. Freiburghaus *et al.*, "Low convergent validity of [11C]raclopride binding in extrastriatal brain regions: A PET study of within-subject correlations with [11C]FLB 457," *Neuroimage*, vol. 226, p. 117523, Feb. 2021, doi: 10.1016/j.neuroimage.2020.117523.
[25] D. Rebelo, F. Oliveira, A. Abrunhosa, C. Januário, J. Lemos, and M. Castelo-Branco, "A link between synaptic plasticity and reorganization of brain activity in Parkinson's disease," Proceedings of the National Academy of Sciences, vol. 118, no. 3, Jan. 2021, doi: 10.1073/pnas.2013962118.

For peer review only

[26] V. L. Darcey *et al.*, "Striatal dopamine tone is positively associated with body mass index in humans as determined by PET using dual dopamine type-2 receptor antagonist tracers", doi: 10.1101/2023.09.27.23296169.
[27] N. Karalija *et al.*, "Longitudinal Dopamine D2 Receptor Changes and Cerebrovascular Health in Aging," *Neurology*, vol. 99, no. 12, pp. e1278–e1289, Sep. 2022, doi: 10.1212/wnl.0000000000200891.
[28] E. Hidalgo-Lopez and B. Pletzer, "Interactive Effects of Dopamine Baseline Levels and Cycle Phase on Executive Functions: The Role of Progesterone," *Front Neurosci*, vol. 11, no. JUL, Jul. 2017, doi: 10.3389/fnins.2017.00403.
[29] P. P. P. Machado, C. Martins, A. R. Vaz, E. Conceição, A. P. Bastos, and S. Gonçalves, "Eating Disorder Examination Questionnaire: Psychometric Properties and Norms for the Portuguese Population," *European Eating Disorders Review*, vol. 22, no. 6, pp. 448–453, Nov. 2014, doi: 10.1002/erv.2318.
[30] P. A. S. Duarte, L. Palmeira, and J. Pinto-Gouveia, "The Three-Factor Eating Questionnaire-R21: a confirmatory factor analysis in a Portuguese sample," *Eating and Weight Disorders - Studies on Anorexia, Bulimia and Obesity*, vol. 25, no. 1, pp. 247–256, Feb. 2020, doi: 10.1007/s40519-018-0561-7.
[31] P. Castilho, J. Pinto-Gouveia, and J. Duarte, "Evaluating the Multifactor Structure of the Long and Short Versions of the Self-Compassion Scale in a Clinical Sample," *J Clin Psychol*, vol. 71, no. 9, pp. 856–870, Sep. 2015, doi: 10.1002/jclp.22187.
[32] S. A. Eisenstein *et al.*, "Insulin, Central Dopamine D2 Receptors, and Monetary Reward Discounting in Obesity," *PLoS One*, vol. 10, no. 7, p. e0133621, Jul. 2015, doi: 10.1371/journal.pone.0133621.
[33] S. A. Eisenstein *et al.*, "Emotional Eating Phenotype is Associated with Central Dopamine D2 Receptor Binding Independent of Body Mass Index," *Sci Rep*, vol. 5, no. 1, p. 11283, Sep. 2015, doi: 10.1038/srep11283.
[34] A. Toet *et al.*, "CROCUFID: A Cross-Cultural Food Image Database for Research on Food Elicited Affective Responses," *Front Psychol*, vol. 10, no. JAN, Jan. 2019, doi: 10.3389/fpsyg.2019.00058.
[35] D. Lakens, "Equivalence Tests," *Soc Psychol Personal Sci*, vol. 8, no. 4, pp. 355–362, May 2017, doi: 10.1177/1948550617697177.
[36] G. Ribeiro, A. Maia, G. Cotovio, F. P. M. Oliveira, D. C. Costa, and A. J. Oliveira-Maia, "Striatal dopamine D2-like receptors availability in obesity and its modulation by bariatric surgery: a systematic review and meta-analysis," *Sci Rep*, vol. 13, no. 1, p. 4959, Mar. 2023, doi: 10.1038/s41598-023-31250-2.

For peer review only

AUTHORS' CONTRIBUTIONS

MLP, JC and MCB designed the protocol of the study. JC and MCB designed the protocol of the MRI experiment. MLP and JC initiated the acquisition of data. MCB supervised the project and provided the funding for PET and MRI acquisition, and AA provided the expertise for [^{11}C]raclopride synthesis. MLP wrote the first draft of the manuscript. MLP, JC, AA and MCB critically revised the manuscript and approved the final version.

FUNDING

This Study Research Proposal was funded in 2020 by *Fundação para a Ciência e a Tecnologia* (FCT) for four years PhD Research Scholarship. Institutional funding is secured by Coimbra Institute for Biomedical Imaging and Translational Research (CIBIT) Funding (FCT/UIDP&B/4950/2020), CENTRO-01-0145-FEDER-000016, DSAIPA/DS/0041/2020 and ICNAS/ICNAS Pharma.

COMPETING INTERESTS STATEMENT

The authors have no competing interests.

Figure Legends

Figure 1 Main and secondary study designs, research questions and samples. Controls are the age- and gender-matched normal-weight healthy individuals.

Figure 2 Block design representing one of the six runs of the fMRI paradigm. Each run is comprised of three visual blocks and three question blocks. The visual blocks are 20 seconds each and are composed of 5 pictures that change every 4 seconds. The first block switches between high- and low-calorie foods, while the block of non-food objects is interleaved in between. Before each visual block takes place a fixation cross (10 seconds) and after a question block (10 seconds), with a question about the visual block that took place before. Food visual blocks will be followed by a decision-making question of whether the person would eat such foods, considering both the pleasure and health implications. On the other hand, non-food visual blocks will be followed by an attention question of whether the person recognises such objects. Cal = calorie; min = minutes; sec = seconds.

For peer review only

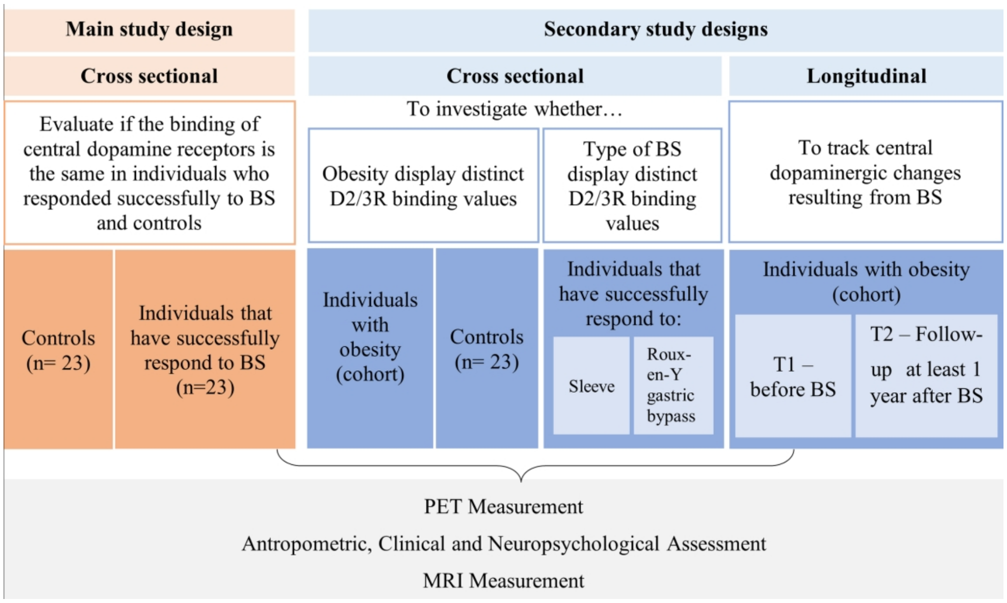

Figure 1 Main and secondary study designs, research questions and samples. Controls are the age- and gender-matched normal-weight healthy individuals.

197x117mm (300 x 300 DPI)

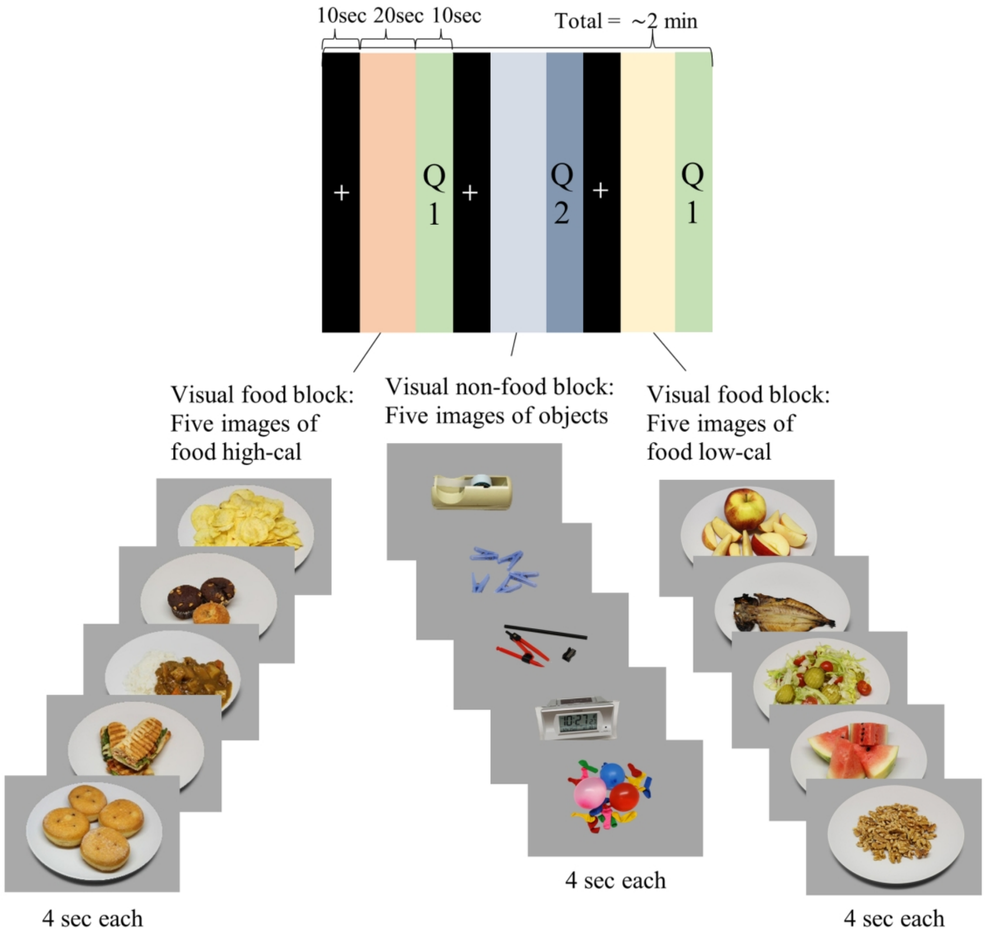

Figure 2 Block design representing one of the six runs of the fMRI paradigm. Each run is comprised of three visual blocks and three question blocks. The visual blocks are 20 seconds each and are composed of 5 pictures that change every 4 seconds. The first block switches between high- and low-calorie foods, while the block of non-food objects is interleaved in between. Before each visual block takes place a fixation cross (10 seconds) and after a question block (10 seconds), with a question about the visual block that took place before. Food visual blocks will be followed by a decision-making question of whether the person would eat such foods, considering both the pleasure and health implications. On the other hand, non-food visual blocks will be followed by an attention question of whether the person recognises such objects. Cal = calorie; min = minutes; sec = seconds.

170x165mm (300 x 300 DPI)

BMJ Open

Portuguese observational cross-sectional clinical imaging study protocol to investigate central dopaminergic mechanisms of successful weight loss through bariatric surgery

Journal:	*BMJ Open*
Manuscript ID	bmjopen-2023-080702.R3
Article Type:	Protocol
Date Submitted by the Author:	13-Mar-2024
Complete List of Authors:	Pais, Marta Lapo; University of Coimbra<br>Crisóstomo, Joana; University of Coimbra<br>Abrunhosa, Antero; University of Coimbra<br>Castelo-Branco, Miguel; University of Coimbra
**Primary Subject Heading**:	Diabetes and endocrinology
Secondary Subject Heading:	Radiology and imaging
Keywords:	Bariatric Surgery, Obesity, Diagnostic Imaging, DIABETES & ENDOCRINOLOGY, RADIOLOGY & IMAGING

SCHOLARONE™
Manuscripts

Title

Portuguese observational cross-sectional clinical imaging study protocol to investigate central dopaminergic mechanisms of successful weight loss through bariatric surgery

Authors

Marta Lapo Pais[a,b,c], Joana Crisóstomo[a,d], Antero Abrunhosa[a,b] and Miguel Castelo-Branco[a,b,e*]

[a] Coimbra Institute for Biomedical Imaging and Translational Research (CIBIT), University of Coimbra, Coimbra, Portugal

[b] Institute for Nuclear Sciences Applied to Health (ICNAS), University of Coimbra, Coimbra, Portugal

[c] Faculty of Science and Technology, University of Coimbra, Coimbra, Portugal

[d] Champalimaud Research, Champalimaud Foundation, Lisbon, Portugal

[e] Faculty of Medicine, University of Coimbra, Coimbra, Portugal

* Corresponding author (mcbranco@fmed.uc.pt)

For peer review only

ABSTRACT

Introduction

Bariatric surgery (BS) is the treatment of choice for refractory obesity. Although weight loss (WL) reduces the prevalence of obesity-related comorbidities, not all patients maintain it. It has been suggested that central mechanisms involving dopamine receptors may play a role in successful WL. This protocol describes an observational cross-sectional study to test if the binding of central dopamine receptors is similar in individuals who responded successfully to BS and age- and gender-matched normal-weight healthy individuals (controls). As secondary goals, the protocol will investigate if this binding correlates with key parameters such as age, hormonal status, anthropometric metrics, and neurobehavioral scores. Finally, as exploratory goals, we will include a cohort of individuals with obesity before and after BS to explore whether obesity and type of BS

(sleeve gastrectomy and Roux-en-Y gastric bypass) yield distinct binding values and track central dopaminergic changes resulting from BS.

Methods and analysis

To address the major research question of this observational study, positron emission tomography (PET) with [^{11}C]raclopride will be used to map brain dopamine type 2 and 3 receptors (D2/3R) non-displaceable binding potential (BP_{ND}) of individuals who have successfully responded to BS. Mean regional D2/3R BP_{ND} values will be compared to control individuals by two one-sided tests (TOST) approaches. The sample size (23 per group) was estimated to demonstrate the equivalence between two independent group means. In addition, these binding values will be correlated with key parameters to address secondary goals. Finally, for exploratory analysis, these values will be compared within the same individuals (before and after BS) and between individuals with obesity and controls and types of BS.

Ethics and dissemination

The project and informed consent received ethical approval from the Faculty of Medicine (FMUC) and the Coimbra University Hospital (CHUC) ethics committees. Results will be disseminated in international peer-reviewed journals and conferences.

Strengths and limitations of this study

→ Investigating brain dopamine receptors in successful WL through BS will ultimately help clarify how these receptors function in the context of maintenance of WL, decreased food intake and reduced motivational drive to eat after BS;

→ Including an exploratory longitudinal analysis employing functional PET at two different time points, the study will be able to track BS-associated central dopaminergic neurotransmission changes;

→ Using functionally defined brain regions will increase the statistical power of the study analysis by defining a mean activation cluster related to visual food cue processing;

→This study was designed to include only female participants, which may limit the generalisability of our results.

→ Exploratory subgroup analysis is limited due to the dataset size, and some level of participant dropout or exclusion is expected in the longitudinal part of this analysis.

For peer review only

Abbreviations

BMI = Body Mass Index; BP_{ND} = nondisplaceable binding potential; BS = bariatric surgery; CHUC = Coimbra Hospital and University Centre; DASS= Depression Anxiety and Stress Scale; D2R = dopamine type 2 receptors; D2/3R = dopamine type 2 and 3 receptors; EDE-Q= Eating Disorder Examination Questionnaire; TE= echo time; EWL% = excess weight loss; FOV= field of view; FA= flip angle; fMRI = functional magnetic resonance imaging; GLM = General Linear Model; ICNAS = Institute for Nuclear Sciences Applied to Health; MRI = magnetic resonance imaging; PET = positron emission tomography; TOST = two one-sided tests; TR= repetition time; ROI = region of interest; SCS= Self-Compassion Scale; TEFQ= Three Eating Factor Questionnaire; WL% = total body weight loss; WL = weight loss.

INTRODUCTION

Background

Obesity is a multifactorial condition thought to result from gene-environment interactions and is mediated by complex neuronal and hormonal mechanisms.[1], [2] As a result, an imbalance of energy where energy intake exceeds energy expenditure leads to weight gain.[1], [2] This condition has become an epidemic of global proportions, being one of the leading causes of morbidity and mortality for the current and likely future generations.[3]

Bariatric surgery (BS) is the treatment of choice when other options have failed to treat obesity. Although weight loss (WL) reduces the prevalence of obesity-related comorbidities and improves the quality of life, not all patients manage to lose weight, and others regain it in the long term.[4], [5], [6] It has been suggested that WL after BS is likely due to changes in central nervous hunger and satiety control rather than purely restrictive gastric volume or gastrointestinal malabsorption.[7] This implies the existence of neural mechanisms underlying BS and the role of central neurotransmission in hunger and satiety control.[8], [9], [10], [11] Dopamine is an important neurotransmitter with implications in addiction as well as WL, decreased food intake and a reduced motivational drive to eat.[12], [13], [14], [15] Concerning cerebral control of food intake, the dopamine type 2 receptors (D2R) are particularly interesting. Original studies on this topic have been reporting that drugs that block D2R increase appetite and result in weight gain, such

For peer review only

as antipsychotic drugs[16] and treatment with dopamine receptor agonists with a high affinity for D2R causes WL.[17] Moreover, a study on diet manipulation found that dietary fat restriction significantly decreased D2R availability in striatal clusters of individuals with obesity.[18] Yet, the role of brain dopamine receptors in eating behaviour that consequently impacts body weight, particularly their function in the context of successful WL through BS, is neither consensual nor well addressed. For this reason, we propose to further investigate these receptors in individuals who have successfully responded to BS.

Study aims

This protocol describes an observational cross-section study whose main goal is to investigate how brain dopamine type 2 and 3 receptors (D2/3R) function in the context of successful WL through BS. Namely, we aim to understand if the binding of these receptors is the same in individuals who responded successfully to BS and controls, age- and gender-matched normal-weight healthy individuals. To achieve that, positron emission tomography (PET) with [^{11}C]raclopride will be used to map the binding of these receptors in 23 individuals of both groups. Mean regional D2/3R non-displaceable binding potential (BP_{ND}) values will be extracted from these images, with a focus on three striatal brain regions (caudate, putamen and ventral striatum) and a mean activation cluster across individuals related to visual food cue processing identified by functional magnetic resonance imaging (fMRI).

As secondary goals, we aim to evaluate if these binding values correlate with anthropometric metrics, including waist/hip circumference, body mass index (BMI), excess weight loss (EWL%), total body weight loss (WL%) and weight regain, and key variables such as neurobehavioral scores, age and hormonal status.

We will also perform an exploratory analysis using the same molecular imaging dopaminergic system. We will compare mean regional D2/3R BP_{ND} values of independent groups of individuals (sleeve gastrectomy and Roux-en-Y gastric bypass, controls and individuals with obesity) to investigate whether the type of BS and obesity display distinct dopamine receptors binding values, respectively. Finally, we will track central dopaminergic changes resulting from BS by comparing the same individuals before and after BS.

For peer review only

We expect this work will help clarify how central mechanisms function in the context of maintenance of WL, decreased food intake and reduced motivational drive to eat after BS. Simultaneously exploring these central mechanisms in obese phenotypes and investigating the differences between types of BS, we can contribute to understanding if the binding of these receptors depends on BMI or the type of surgery used.

METHODS AND ANALYSIS

Study design

This study can be split into main and secondary designs. The main design will be used to answer the major and secondary questions, whereas the secondary design will be used for exploratory research questions (see Figure 1). Both studies will be conducted at the Institute for Nuclear Sciences Applied to Health (ICNAS) University of Coimbra, Coimbra, Portugal. The expected overall study duration is approximately three years, from January 2022 (when we obtained both approvals from ethics committees) to January 2025. We anticipate a three-year timeframe to disseminate the study in the community and social media, collect contacts of possible participants, optimise and test the study protocol by pilot acquisitions using healthy volunteers from our volunteer database, recruit participants, acquire and analyse the data and submit the results for publication in peer-reviewed journals.

The main study design is an observational cross-sectional clinical imaging study to evaluate if the binding of D2/3R is the same in individuals who responded successfully to BS and controls. Two groups of female subjects will be included: women who have successfully responded to BS (n=23) recruited from Coimbra Hospital and University Centre (CHUC) or the Portuguese association of patients who underwent BS – *Associação Portuguesa dos Bariátricos* (APOBARI); and age- and gender-matched normal-weight (BMI: 18.5-24.9 Kg/m^2) healthy individuals (n= 23) recruited from our volunteer database and public advertisements. To be successful BS responders, individuals should achieve at least 50% EWL% or 20% of WL% at 1-year post-op.[19] Since we will include participants from 1-5 years post-op, we will use a retrospective approach to verify this criterion and consider the weight participants had 1-year post-op. We will also guarantee that at the time of data acquisition, participants still fulfil the

For peer review only

criterion defined for 1 year after surgery (> 50% EWL% or 20% TWL%). Follow-up time post-op will be further used as a covariate in the statistical analysis.

The secondary study design includes a longitudinal and a cross-sectional part. The longitudinal part was designed to track central dopaminergic changes resulting from BS. For that purpose, we will include a cohort of individuals with obesity at two different time points, before and after BS. On the other hand, in the cross-sectional part, we will split the individuals who underwent BS according to the type of surgery to investigate whether the type of BS displays distinct dopamine receptor binding values. Finally, to investigate whether these binding values are altered in obesity, we will compare the individuals with obesity of the cohort with the controls. The study designs, research questions, and samples are schematised in Figure 1.

Study-specific inclusion criteria are signed informed consent, women aged between 18 and 65 years, BMI >33 kg/m^2 for individuals with obesity and 18.5-24.9 Kg/m^2 for age- and gender-matched normal-weight healthy individuals. No restriction regarding BMI range will be imposed on the group who have successfully responded to BS. Exclusion criteria are previous gastrointestinal surgery, current treatment with medication interacting with central dopamine, active neoplastic or inflammatory disease, history of neurological disease, traumatic brain injury or psychiatric disorder, previous/current alcohol or other substance abuse and conditions that preclude magnetic resonance imaging (MRI) or PET.

Patient and public involvement

Patients were not involved in the development of the research question, outcome measures or study design but were involved in the dissemination.

PET Measurement (acquisition and analysis)

Dynamic PET scan of the entire brain in 3D mode for 60 min (24 frames: 4x15sec, 4x30sec, 3x60sec, 2x120s, 5x240, 6x300s) together with computed tomography after intravenous bolus injection of a maximum of 15mCi of [^{11}C]raclopride will be acquired using PET/CT scanner (Siemens Biograph Vision 600). A dedicated and experienced team will perform [^{11}C]raclopride synthesis in the facilities of the ICNAS according to the methods described in Rodrigues et al. (2020).[20]

For peer review only

After acquisition, accurate co-registration (between PET images and their anatomical MRI images) will be performed and further confirmed by visual inspection across all planes. As binding of [^{11}C]raclopride is negligible in the cerebellum, simplified reference tissue models will be used to analyse PET images with [^{11}C]raclopride.[21] As a result, we will obtain the parametric maps of D2/3R BP_{ND}. These maps will be generated using the cerebellum as a reference region, and two distinct reference tissue methods will be evaluated that are Logan Plot and MRTM2 (multilinear reference tissue model 2), as described and evaluated by the methods thoroughly reported in Logan (2000)[22] and Ichise et al. (2003)[23], respectively. Mean regional D2/3R BP_{ND} values of anatomical brain regions of interest (ROIs) will be extracted from the parametric maps. Due to the low signal-to-noise ratio of [^{11}C]raclopride in extrastriatal brain regions[24], anatomical ROIs will be defined in three subregions of the striatum, caudate, putamen and ventral striatum. Additionally, to increase the statistical power of the study analysis, we will test a mean activation cluster resulting from fMRI analysis, which will allow us to take into account other regions where dopaminergic receptors play an important biological role.[25] All PET data analysis will be performed using the Pmod software (PMOD, version 4.105; PMOD Technologies; Zurich, Switzerland) and SPM12 (Version 12, Wellcome Trust Centre for Neuroimaging, London, UK).

Despite the lack of studies, we expect the same mean regional D2/3R BP_{ND} values in individuals who have successfully responded to BS and healthy controls. We hypothesise that D2/3R BP_{ND} depend on the motivation drive to eat, which we speculate to be the same in these two groups. Following the same line of thought, in exploratory analysis, we expect that obesity will be associated with altered mean regional D2/3R BP_{ND} values in the brain but no differences between types of BS (sleeve gastrectomy and roux-en-Y gastric bypass). Moreover, we expect that these dysfunctional values can be reversed to control condition values after WL. It is important to note that because [^{11}C]raclopride is susceptible to endogenous dopamine, D2/3R BP_{ND} values do not reflect only receptor availability but also the levels of dopamine tone. A recent preprint study compared [^{11}C]raclopride to [^{18}F]fallypride, a radiotracer also susceptible to endogenous dopamine but with a higher affinity for D2/3R, and concluded that D2/3R BP_{ND} values measured by PET with [^{11}C]raclopride are more sensitive for revealing between-group differences resulting from dopamine tone.[26]

For peer review only

Clinical and Neuropsychological Assessment

The key variables of the clinical assessment include age, five anthropometric metrics and hormonal status. Anthropometric metrics are BMI, waist/hip circumference ratio for all groups and EWL%, WL% and weight regain for the group of individuals who underwent BS. As all our participants are female, and since dopaminergic neurotransmission might fluctuate across age[27] and the menstrual cycle[28], this information will be further used as a covariate in the statistical analysis. A brief neuropsychological evaluation will also be performed using the Eating Disorder Examination Questionnaire (EDE-Q) of 28 items[29], the Three Eating Factor Questionnaire (TEFQ) of 21 items[30], the Self-Compassion Scale (SCS) of 12 items[31] and the Depression Anxiety and Stress Scale (DASS) of 21 items, all translated and validated for the Portuguese population. EDE-Q has 4 subscales (Restraint, Eating Concern, Shape Concern and Weight Concern) and an overall global score. TEFQ includes three (Cognitive Restraint, Uncontrolled Eating and Emotional Eating). SCS is split into 6 subscales (Self-kindness, Self-judgement, Common Humanity, Isolation, Mindfulness and Overidentification) and an overall global score. Finally, DASS measure the emotional states of depression, anxiety and stress. To limit the number of parameters under investigation, for EDE-Q and SCS, we will only include the overall global score.

Authors have suggested that emotional eating or behaviour of discounting monetary rewards might provide a more accurate reflection of changes in central D2R function compared to BMI[32], [33]. Following this line of thought and our hypothesis - D2/3R BP_{ND} depend on the motivation drive to eat - we expect that neuropsychological scores correlate more strongly with central dopamine receptor function than anthropometric metrics. Namely, we expect to obtain a negative correlation between measures of dysfunctional eating behaviour/concern and self-compassion, depression, anxiety, stress and regional D2/3R BP_{ND} values and no correlation with anthropometric metrics.

MRI Measurement (acquisition and analysis)

Structural and fMRI data will be acquired on a 3T imaging system (MAGNETOM Prisma, Siemens Medical Solutions) using a 64-channel head-coil. First, we will acquire the structural MRI data using 3D-T1 weighted sequence (192 slices; echo time (TE): 3.5 ms; repetition time (TR): 2530 ms; voxel size: 1×1×1 mm; flip angle (FA): 7°; field

For peer review only

of view (FOV): 256×256 mm). After that, we will start the block-design fMRI paradigm (6 runs of ~2 minutes each) using 2D-T2* weighted sequences (72 interleaved slices; TE: 37ms; TR: 1000 ms; voxel size: 2×2×2 mm; FA: 68°; FOV: 200×200 mm). Each run comprises three visual blocks and three question blocks. In each visual block (20 seconds), participants will be presented with 5 images of low or high-calorie foods or non-food objects from the Cross Cultural Food Images Database (CROCUFID).[34] For all participants, the sequence of the fMRI task is the same, where the first block switches between high- and low-calorie foods, while the block of non-food objects is interleaved in the middle. Before each visual block takes place a fixation cross (10 seconds) and after a question block (10 seconds), with a question about the visual block that took place before. Food visual blocks will be followed by a decision-making question of whether the person would eat such foods, considering both the pleasure and health implications. To answer the question, the participant will use a response button to choose one of four options: "1- I certainly wouldn't eat it", "2- I probably wouldn't eat", "3- I would probably eat" and "I would certainly eat it". On the other hand, non-food visual blocks will be followed by an attention question of whether the person recognises such objects. Here, the options are the following: "1- I recognise all objects", "2- I recognise almost all objects"; "3-I hardly recognise any object" and "4- I don't recognise any objects". See Figure 2 for details. We plan to use the decision-making questions to evaluate how people rate rewards in terms of pleasure and health concerns, and in particular to achieve integrated information on psychological components of reward, particularly liking (hedonic impact) and wanting (incentive salience). This information will be combined with the neuropsychological scores to conduct a correlation analysis with central dopamine receptor function. Finally, after the fMRI acquisition, the participants will also perform rating tasks using a horizontal, 100-mm, bidirectional scale between the extremes "I don't like it at all" and "I like it a lot" to rate all the food images from fMRI visual task in terms of preferences regardless of health concerns. This information will also be used as a variable in the correlation analysis with central dopamine receptor function.

As was mentioned before, fMRI data using visual stimuli will be used to obtain a mean activation cluster related to visual food cue processing to use as mask in the quantitative analysis of PET brain images. Pre-processing of fMRI data includes head motion correction, mean intensity adjustment, cubic spine slice scan time correction, trilinear 3D motion correction and temporal filtering (high pass). Runs exceeding 3 mm of movement

For peer review only

in any axis will be excluded from further analysis. After inhomogeneity correction of structural MRI data, functional and structural MRI data will be coregistered and transformed to the standard Montreal Neurological Institute (MNI) space. A random effects General Linear Model (GLM) analysis will be computed for each subject to assess individual brain activity patterns. After that, to obtain activation clusters related to visual food cue processing, we will use contrasts between [High-calorie], [Low-calorie] and [Non-food objects]. Finally, we will aggregate the individual activation clusters in a mean activation cluster across groups to use as a mask in the PET analysis. Due to the low signal-to-noise ratio of [^{11}C]raclopride in extrastriatal brain regions[24], we plan to be cautious while not ignoring that D2R are present with meaningful biological function outside the striatum.[25] All MRI data analysis will be performed using BrainVoyager 22.0.0 (Brain Innovation, Maastricht, The Netherlands).

Setting for imaging acquisition (PET and MRI)

PET and MRI acquisition will be performed on the same day by dedicated and experienced technicians in the facilities of ICNAS. Due to the well-documented impact of the fasted vs. fed state on responses to food cues, all subjects will perform MRI acquisition at the same time of the day, around 13:30 in a 6-hour (at least) fast state. To do so, participants will be instructed to have their last meal around 7:30. After the MRI scan, participants will consume the same standardised meal consisting of a vegetable soup, a sandwich, and fruit to ensure a similar recent dietary intake for all participants. For PET acquisition, participants will be instructed to abstain from alcohol for 24 hours, as well as from nicotine and caffeine for 12 hours before this acquisition.

Statistical analyses

As already mentioned, mean regional binding values will be extracted from the D2/3R BP_{ND} parametric maps of 23 individuals who have successfully responded to BS and 23 normal-weight healthy control individuals. Two one-sided tests (TOST) approaches will be performed to investigate if the binding of these receptors is the same in these two groups. To deal with multiple comparison issues, we will limit our hypotheses to three anatomical subregions of the striatum (caudate, putamen and ventral striatum) and a mean activation cluster across groups.

For peer review only

Since correlation is usually tested for two variables at a time, the association between the mean regional D2/3R BP_{ND} values with anthropometric, neurobehavioral and clinical parameters will lead us to the same multiple comparison issues. Since we aim to test several variables, we will perform a correlation matrix where the mean regional D2/3R BP_{ND} values of the three ROIs and the mean activation cluster across groups will be correlated with the age, hormonal status, 5 anthropometric metrics, neuropsychological scores, and fMRI decision-making question responses. The generated matrix will allow us to see which pairs correlate most.

Finally, the exploratory analysis will compare independent groups of individuals to verify if obesity and the type of BS (sleeve gastrectomy and Roux-en-Y gastric bypass) display distinct mean regional D2/3R BP_{ND} values. Since we are comparing independent groups of individuals, we will use multiple unpaired t-tests (for equal variance) or Welch's t-test (for unequal variance) followed by the Holm-Sidak method (to deal with multiple comparison issues). On the other hand, to track central dopaminergic changes resulting from BS, we will compare the same individuals before and after BS using ANOVA: Repeated measures, within-between interaction (for equal variance) or mixed-effects models (for unequal variance). We will perform Dunn's post hoc test to deal with multiple comparison issues in this case.

Sample size justification

The sample size of 23 per group was estimated using the equation (5) published in Lakens (2017) as the required to demonstrate the equivalence between two independent group means for an allocation ratio of 1, equivalence bound of 0.8236, α level of 0.05 and power of 80%.[35] The equivalence bound of 0.8236 was calculated by multiplying 1.16 (Cohen's d) with 0.71 (pooled standard deviation). These values were set based on a meta-analysis performed to investigate group differences in case–control studies comparing the same central dopamine receptors between individuals with obesity and non-obese controls.[36] In the case of significant differences, the overall Cohen's d was -1.16 $\pm$ 0.71.

ETHICS AND DISSEMINATION

The project has received ethical approval from the local medical ethics committee of the Faculty of Medicine of the University of Coimbra (FMUC – approval ID, CE_Proc. CE-

088/202) and Coimbra Hospital and University Centre (CHUC, approval ID OBS.SF.143/2021). Participants will sign an informed consent approved by these ethics committees. Results will be submitted for publication in international peer-reviewed journals and presented at relevant conferences. Summaries of the results will be provided to the participants.

REFERENCES

[1] K. Pak, S. J. Kim, and I. J. Kim, "Obesity and Brain Positron Emission Tomography," *Nucl Med Mol Imaging*, vol. 52, no. 1, pp. 16–23, 2018, doi: 10.1007/s13139-017-0483-8.

[2] G. Ribeiro, M. Camacho, O. Santos, C. Pontes, S. Torres, and A. J. Oliveira-Maia, "Association between hedonic hunger and body-mass index versus obesity status," *Sci Rep*, vol. 8, no. 1, pp. 1–9, 2018, doi: 10.1038/s41598-018-23988-x.

[3] B. J. Sargent and A. J. Henderson, "Targeting 5-HT receptors for the treatment of obesity," *Curr Opin Pharmacol*, vol. 11, no. 1, pp. 52–58, 2011, doi: 10.1016/j.coph.2011.01.005.

[4] M. C. Gulliford *et al.*, "Costs and outcomes of increasing access to bariatric surgery for obesity: cohort study and cost-effectiveness analysis using electronic health records," *Health Services and Delivery Research*, vol. 4, no. 17, pp. 1–120, 2016, doi: 10.3310/hsdr04170.

[5] Z. Khorgami, S. Shoar, A. Andalib, A. Aminian, S. A. Brethauer, and P. R. Schauer, "Trends in utilization of bariatric surgery, 2010-2014: sleeve gastrectomy dominates," *Surgery for Obesity and Related Diseases*, vol. 13, no. 5, pp. 774–778, 2017, doi: 10.1016/j.soard.2017.01.031.

[6] M. E. Haahr *et al.*, "Central 5-HT Neurotransmission Modulates Weight Loss following Gastric Bypass Surgery in Obese Individuals," *Journal of Neuroscience*, vol. 35, no. 14, pp. 5884–5889, Apr. 2015, doi: 10.1523/JNEUROSCI.3348-14.2015.

[7] H. Schlögl, A. Horstmann, A. Villringer, and M. Stumvoll, "Functional neuroimaging in obesity and the potential for development of novel treatments," *Lancet Diabetes Endocrinol*, vol. 4, no. 8, pp. 695–705, 2016, doi: 10.1016/S2213-8587(15)00475-1.

[8] M. K. Hankir, F. Seyfried, A. D. Miras, and M. A. Cowley, "Brain Feeding Circuits after Roux-en-Y Gastric Bypass," *Trends in Endocrinology & Metabolism*, vol. 29, no. 4, pp. 218–237, Apr. 2018, doi: 10.1016/j.tem.2018.01.009.

[9] K. A. Galen, K. W. Horst, and M. J. Serlie, "Serotonin, food intake, and obesity," *Obesity Reviews*, vol. 22, no. 7, pp. 1–13, Jul. 2021, doi: 10.1111/obr.13210.

[10] A. J. Oliveira-Maia, C. D. Roberts, S. A. Simon, and M. A. L. Nicolelis, "Gustatory and reward brain circuits in the control of food intake," in *Psychiatry: Interpersonal and Biological Processes*, 2011, pp. 31–59. doi: 10.1007/978-3-7091-0179-7_3.

[11] M. A. Rossi and G. D. Stuber, "Overlapping Brain Circuits for Homeostatic and Hedonic Feeding," *Cell Metab*, vol. 27, no. 1, pp. 42–56, Jan. 2018, doi: 10.1016/j.cmet.2017.09.021.

For peer review only - http://bmjopen.bmj.com/site/about/guidelines.xhtml

[12] S. E. Simonds and M. A. Cowley, "Speed-dieting: dopamine agonists promote weight loss," *Nat Metab*, vol. 1, no. 9, pp. 851–852, Aug. 2019, doi: 10.1038/s42255-019-0114-z.

[13] K. A. van Galen, K. W. ter Horst, J. Booij, S. E. la Fleur, and M. J. Serlie, "The role of central dopamine and serotonin in human obesity: lessons learned from molecular neuroimaging studies," *Metabolism*, vol. 85, pp. 325–339, Aug. 2018, doi: 10.1016/j.metabol.2017.09.007.

[14] S. Hesse *et al.*, "Central serotonin transporter availability in highly obese individuals compared with non-obese controls: A [11C] DASB positron emission tomography study," *Eur J Nucl Med Mol Imaging*, vol. 43, no. 6, pp. 1096–1104, Jun. 2016, doi: 10.1007/s00259-015-3243-y.

[15] L. K. Janssen and A. Horstmann, "Molecular Imaging of Central Dopamine in Obesity: A Qualitative Review across Substrates and Radiotracers," *Brain Sci*, vol. 12, no. 4, p. 486, Apr. 2022, doi: 10.3390/brainsci12040486.

[16] M.-J. Poulin et al., "Management of Antipsychotic-Induced Weight Gain: Prospective Naturalistic Study of the Effectiveness of a Supervised Exercise Programme," Australian & New Zealand Journal of Psychiatry, vol. 41, no. 12, pp. 980–989, Dec. 2007, doi: 10.1080/00048670701689428.

[17] C. Folgueira *et al.*, "Hypothalamic dopamine signalling regulates brown fat thermogenesis," *Nat Metab*, vol. 1, no. 8, pp. 811–829, Aug. 2019, doi: 10.1038/s42255-019-0099-7.

[18] V. L. Darcey *et al.*, "Dietary fat restriction affects brain reward regions in a randomized crossover trial," *JCI Insight*, vol. 8, no. 12, Jun. 2023, doi: 10.1172/jci.insight.169759.

[19] S. Sanchez-Cordero *et al.*, "Analysis of the Variability in Different Criteria to Define the Success of Bariatric Surgery: Retrospective Study 5-Year Follow-Up after Sleeve Gastrectomy and Roux-en-Y Gastric Bypass," *J Clin Med*, vol. 12, no. 1, p. 187, Dec. 2022, doi: 10.3390/jcm12010187.

[20] I. Beatriz, A. Rodrigues, F. José, and C. Alves, "Synthesis Optimisation of High Specific Activity Carbon-11 Radiopharmaceuticals for Brain PET Studies," 2020.

[21] Turku PET entre, "Quantification of [carbonyl-11C]WAY-100635 PET." Accessed: Aug. 12, 2021. [Online]. Available: http://www.turkupetcentre.net/petanalysis/analysis_11c-way100635.html

[22] J. Logan, "Graphical analysis of PET data applied to reversible and irreversible tracers," *Nucl Med Biol*, vol. 27, no. 7, pp. 661–670, Oct. 2000, doi: 10.1016/S0969-8051(00)00137-2.

[23] M. Ichise *et al.*, "Linearized reference tissue parametric imaging methods: Application to [11C]DASB positron emission tomography studies of the serotonin transporter in human brain," *Journal of Cerebral Blood Flow and Metabolism*, vol. 23, no. 9, pp. 1096–1112, 2003, doi: 10.1097/01.WCB.0000085441.37552.CA.

[24] T. Freiburghaus *et al.*, "Low convergent validity of [11C]raclopride binding in extrastriatal brain regions: A PET study of within-subject correlations with [11C]FLB 457," *Neuroimage*, vol. 226, p. 117523, Feb. 2021, doi: 10.1016/j.neuroimage.2020.117523.

[25] D. Rebelo, F. Oliveira, A. Abrunhosa, C. Januário, J. Lemos, and M. Castelo-Branco, "A link between synaptic plasticity and reorganization of brain activity in Parkinson's disease," Proceedings of the National Academy of Sciences, vol. 118, no. 3, Jan. 2021, doi: 10.1073/pnas.2013962118.

For peer review only

[26] V. L. Darcey *et al.*, "Striatal dopamine tone is positively associated with body mass index in humans as determined by PET using dual dopamine type-2 receptor antagonist tracers", doi: 10.1101/2023.09.27.23296169.
[27] N. Karalija *et al.*, "Longitudinal Dopamine D2 Receptor Changes and Cerebrovascular Health in Aging," *Neurology*, vol. 99, no. 12, pp. e1278–e1289, Sep. 2022, doi: 10.1212/wnl.0000000000200891.
[28] E. Hidalgo-Lopez and B. Pletzer, "Interactive Effects of Dopamine Baseline Levels and Cycle Phase on Executive Functions: The Role of Progesterone," *Front Neurosci*, vol. 11, no. JUL, Jul. 2017, doi: 10.3389/fnins.2017.00403.
[29] P. P. P. Machado, C. Martins, A. R. Vaz, E. Conceição, A. P. Bastos, and S. Gonçalves, "Eating Disorder Examination Questionnaire: Psychometric Properties and Norms for the Portuguese Population," *European Eating Disorders Review*, vol. 22, no. 6, pp. 448–453, Nov. 2014, doi: 10.1002/erv.2318.
[30] P. A. S. Duarte, L. Palmeira, and J. Pinto-Gouveia, "The Three-Factor Eating Questionnaire-R21: a confirmatory factor analysis in a Portuguese sample," *Eating and Weight Disorders - Studies on Anorexia, Bulimia and Obesity*, vol. 25, no. 1, pp. 247–256, Feb. 2020, doi: 10.1007/s40519-018-0561-7.
[31] P. Castilho, J. Pinto-Gouveia, and J. Duarte, "Evaluating the Multifactor Structure of the Long and Short Versions of the Self-Compassion Scale in a Clinical Sample," *J Clin Psychol*, vol. 71, no. 9, pp. 856–870, Sep. 2015, doi: 10.1002/jclp.22187.
[32] S. A. Eisenstein *et al.*, "Insulin, Central Dopamine D2 Receptors, and Monetary Reward Discounting in Obesity," *PLoS One*, vol. 10, no. 7, p. e0133621, Jul. 2015, doi: 10.1371/journal.pone.0133621.
[33] S. A. Eisenstein *et al.*, "Emotional Eating Phenotype is Associated with Central Dopamine D2 Receptor Binding Independent of Body Mass Index," *Sci Rep*, vol. 5, no. 1, p. 11283, Sep. 2015, doi: 10.1038/srep11283.
[34] A. Toet *et al.*, "CROCUFID: A Cross-Cultural Food Image Database for Research on Food Elicited Affective Responses," *Front Psychol*, vol. 10, no. JAN, Jan. 2019, doi: 10.3389/fpsyg.2019.00058.
[35] D. Lakens, "Equivalence Tests," *Soc Psychol Personal Sci*, vol. 8, no. 4, pp. 355–362, May 2017, doi: 10.1177/1948550617697177.
[36] G. Ribeiro, A. Maia, G. Cotovio, F. P. M. Oliveira, D. C. Costa, and A. J. Oliveira-Maia, "Striatal dopamine D2-like receptors availability in obesity and its modulation by bariatric surgery: a systematic review and meta-analysis," *Sci Rep*, vol. 13, no. 1, p. 4959, Mar. 2023, doi: 10.1038/s41598-023-31250-2.

For peer review only

AUTHORS' CONTRIBUTIONS

MLP, JC and MCB designed the protocol of the study. JC and MCB designed the protocol of the MRI experiment. MLP and JC initiated the acquisition of data. MCB supervised the project and provided the funding for PET and MRI acquisition, and AA provided the expertise for [^{11}C]raclopride synthesis. MLP wrote the first draft of the manuscript. MLP, JC, AA and MCB critically revised the manuscript and approved the final version.

FUNDING

This Study Research Proposal was funded in 2020 by *Fundação para a Ciência e a Tecnologia* (FCT) for four years PhD Research Scholarship. Institutional funding is secured by Coimbra Institute for Biomedical Imaging and Translational Research (CIBIT) Funding (FCT/UIDP&B/4950/2020), CENTRO-01-0145-FEDER-000016, DSAIPA/DS/0041/2020 and ICNAS/ICNAS Pharma.

COMPETING INTERESTS STATEMENT

The authors have no competing interests.

Figure Legends

Figure 1 Main and secondary study designs, research questions and samples. Controls are the age- and gender-matched normal-weight healthy individuals.

Figure 2 Block design representing one of the six runs of the fMRI paradigm. Each run is comprised of three visual blocks and three question blocks. The visual blocks are 20 seconds each and are composed of 5 pictures that change every 4 seconds. The first block switches between high- and low-calorie foods, while the block of non-food objects is interleaved in between. Before each visual block takes place a fixation cross (10 seconds) and after a question block (10 seconds), with a question about the visual block that took place before. Food visual blocks will be followed by a decision-making question of whether the person would eat such foods, considering both the pleasure and health implications. On the other hand, non-food visual blocks will be followed by an attention question of whether the person recognises such objects. Cal = calorie; min = minutes; sec = seconds.

For peer review only

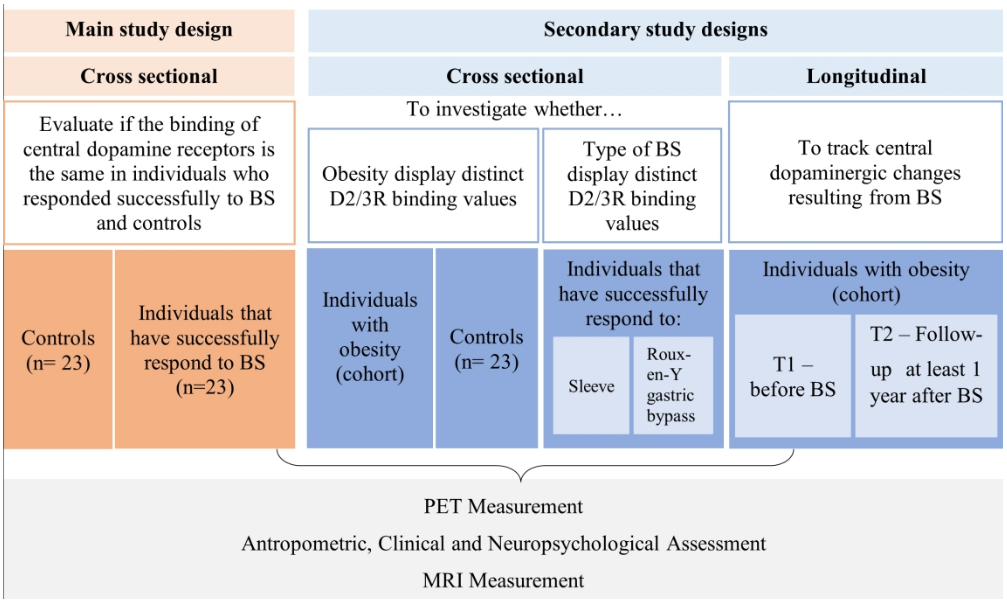

Figure 1 Main and secondary study designs, research questions and samples. Controls are the age- and gender-matched normal-weight healthy individuals.

197x117mm (330 x 330 DPI)

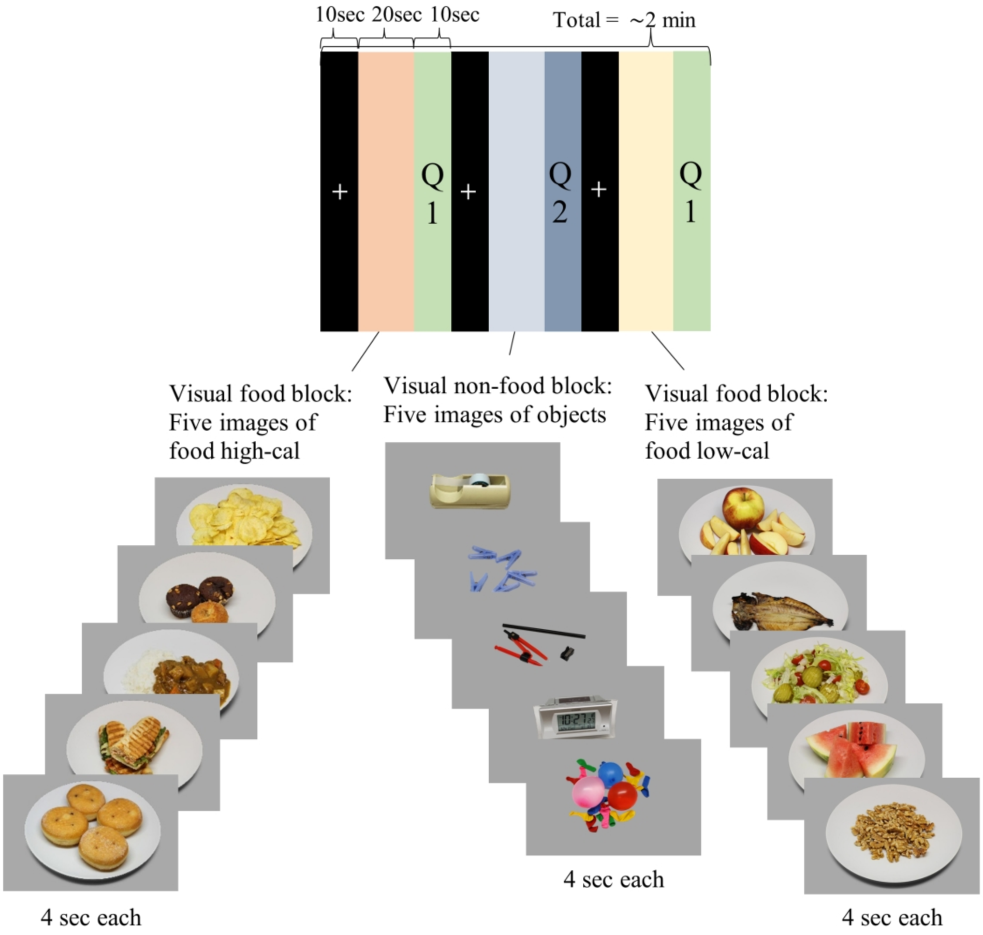

Figure 2 Block design representing one of the six runs of the fMRI paradigm. Each run is comprised of three visual blocks and three question blocks. The visual blocks are 20 seconds each and are composed of 5 pictures that change every 4 seconds. The first block switches between high- and low-calorie foods, while the block of non-food objects is interleaved in between. Before each visual block takes place a fixation cross (10 seconds) and after a question block (10 seconds), with a question about the visual block that took place before. Food visual blocks will be followed by a decision-making question of whether the person would eat such foods, considering both the pleasure and health implications. On the other hand, non-food visual blocks will be followed by an attention question of whether the person recognises such objects. Cal = calorie; min = minutes; sec = seconds.

171x165mm (330 x 330 DPI)
